# Multilevel synergistic mechanisms of a nanocarrier-enabled topramezone herbicide against invasive weeds

**DOI:** 10.1186/s12951-026-04472-5

**Published:** 2026-04-22

**Authors:** Jingyi Chen, Heng Qiao, Xiao Ran, Jixing Xia, Hegan Dong, Hanyue Wang, Huan Peng, Meizhen Yin, Min Dong, Jie Shen, Shuo Yan

**Affiliations:** 1 State Key Laboratory of Agricultural and Forestry Biosecurity, Department of Plant Biosecurity, College of Plant Protection, China Agricultural University, Beijing, 100193 PR China; 2 College of Life Sciences in Shihezi University and Xinjiang Production and Construction Corps Key Laboratory of Oasis Town and Mountain-basin System Ecology, Shihezi, 832003 PR China; 3 Institute of Western Agriculture, Chinese Academy of Agricultural Sciences, Changji, 453500 PR China; 4 State Key Laboratory for Biology of Plant Diseases and Insect Pests, Institute of Plant Protection, Chinese Academy of Agricultural Sciences, Beijing, 100193 PR China; 5 State Key Laboratory of Chemical Resource Engineering, Beijing Lab of Biomedical Materials, Beijing University of Chemical Technology, Beijing, 100029 PR China

**Keywords:** Drug delivery, Foliar uptake, Nano-herbicide, Self-assembly, Invasive weed

## Abstract

The rapid proliferation of invasive weeds has emerged as an escalating “major threat”, and the inefficient utilization and environmental dispersion of conventional herbicides pose persistent challenges in agricultural production. Herein, an amphiphilic hydrophilic–lipophilic diblock polymer (HLDP) was constructed to develop a nano-enabled topramezone (TOP) with excellent herbicidal performance toward invasive weed *Cyclachaena xanthiifolia* while reducing ecological risk. The HLDP displayed a high loading content of 46.22% toward TOP, and the dynamic self-assembly was spontaneously driven by hydrophobic association and hydrogen bonding, characterized by isothermal titration calorimetry, molecular docking/dynamics simulation and Fourier transform infrared spectroscopy. The encapsulation within HLDP decreased the particle size of TOP down to 102 nm to form stable spherical particles with the zeta potential of 30.13 mV, and the thermal stability of TOP was significantly improved by forming a hybrid nanostructure. Compared to free TOP, the TOP@HLDP exhibited excellent foliar behavior with reduced contact angle (1.4-fold), enhanced spray retention (2.3-fold), and increased systemic uptake (2-fold), as well as altered soil transport behavior. Notably, both laboratory and field trials demonstrated significantly enhanced herbicidal activity of TOP@HLDP compared with free and commercial formulations, resulting in stronger growth inhibition and reduced chlorophyll and nitrogen contents. Integrated transcriptomic and metabolomic analyses revealed that the complexation with HLDP further induced amplified oxidative and hormonal stress, characterized by suppressed photosynthetic electron transport, impaired NADPH generation, redirected carbon allocation, and enhanced jasmonic acid biosynthesis, which was consistent with the physiological responses in invasive weeds. Moreover, biosafety assessments confirmed the superior biocompatibility of TOP@HLDP toward non-target organisms, including fruit flies, S2 cells and maize seeds. Collectively, our study extensively elucidated the multiple synergistic mechanisms underlying the HLDP-based delivery system from dynamic self-assembly to multi-omics basis, offering a reliable strategy for designing/developing nano-herbicides with high bioactivity and excellent biocompatibility toward invasive weeds.

**Graphical abstract:**

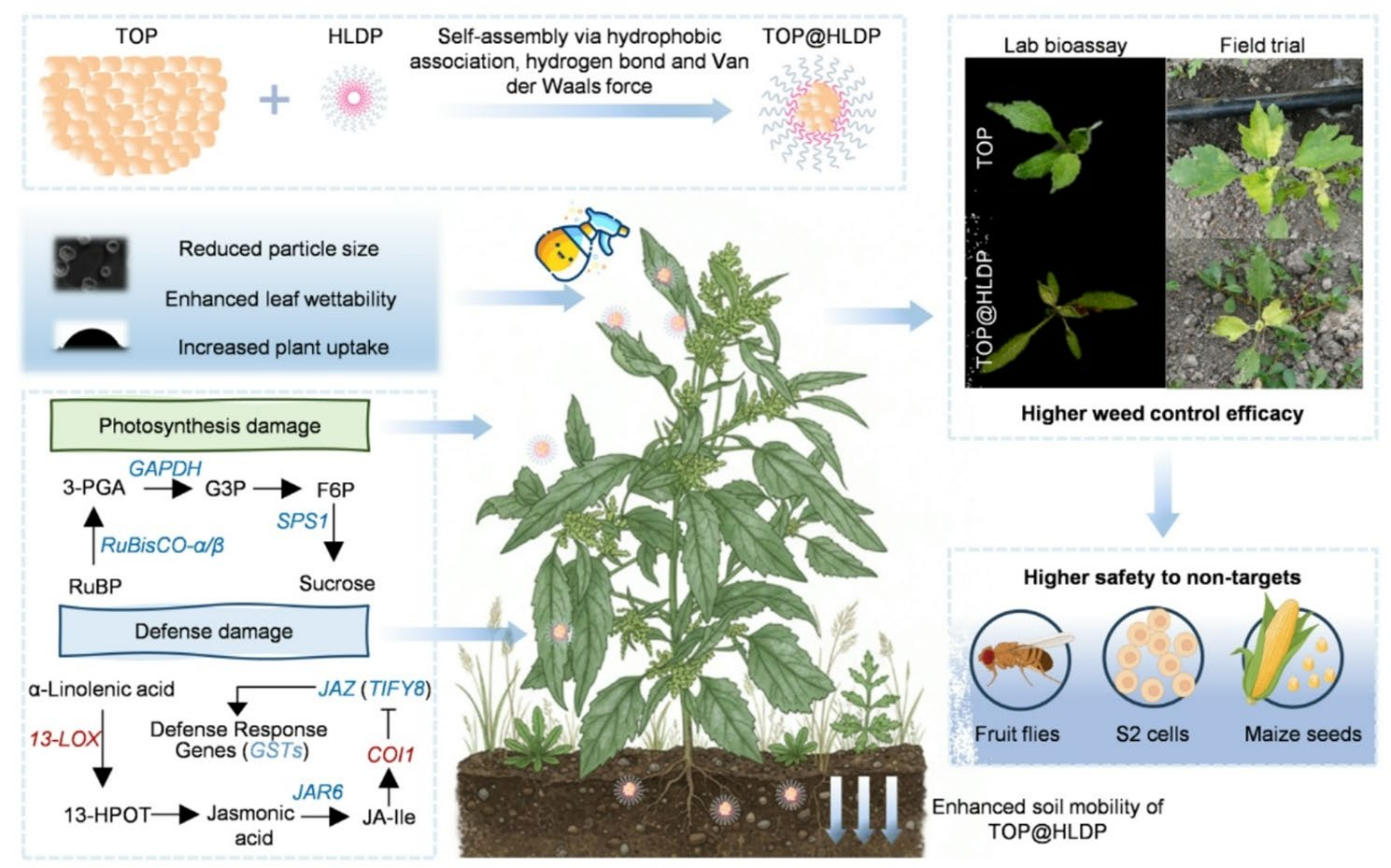

**Supplementary Information:**

The online version contains supplementary material available at 10.1186/s12951-026-04472-5.

## Introduction

Global agricultural resilience is fundamentally undermined by weed infestations, which represent one of the most pervasive and economically devastating biotic constraint to crop yields worldwide [1, 2]. As relentless competitors for vital growth factors, such as solar radiation, aqueous resources, mineral nutrients, and spatial niche, weeds exert a chronic and cumulative suppressive pressure that spans the entire crop ontogeny. Unlike the stochastic/episodic outbreak characteristic of insect pests and phytopathogens, weed infestation constitutes an inherent and sustained competitive antagonism [3, 4]. Approximately 1800 weed species can cause about 30% reduction in crop yields, which translates to USD 30–100 billion economic losses per year [2, 5, 6]. Among harmful weeds, the rapid proliferation of invasive weeds has emerged as an escalating “major threat”; their exceptional phenotypic plasticity and competitive dominance not only jeopardize crop yields, but also catalyze the systemic degradation of both agroecosystems and adjacent biodiversity hotspots [7, 8].

Among the most aggressive invasive weeds, *Cyclachaena xanthiifolia* has emerged as a serious threat to global agroecosystems and biodiversity [9–12]. Originating from North America, this annual herbaceous weed has undergone a rapid transcontinental radiation across Eurasia and now colonizes a wide range of habitats, including croplands and disturbed environments. Its invasive prowess is underpinned by a synergistic combination of pronounced phenotypic plasticity, prolific seed production, and potent allelopathic interference, which collectively facilitate the rapid displacement of native flora and the severe suppression of crop vigor. For example, extracts of *C. xanthiifolia* have been reported to disrupt endogenous hormone balance and antioxidant systems in target plants, thereby inhibiting seed germination and early growth [13]. In addition to agricultural losses, its allergenic pollen and toxic secondary metabolites also pose risks to public health and livestock [14]. In China, *C. xanthiifolia* has shifted from localized occurrence to widespread infestation across major agricultural regions, making it a representative invasive species to increase biosecurity concern. Despite its growing threat, effective control strategies for *C. xanthiifolia* remain limited. As a recently recognized key invasive species in China, only a limited number of registered herbicides are specifically recommended for its control, highlighting an urgent need for improved management approaches. Therefore, rather than investing substantial time and resources in the development of new dedicated herbicides, prioritizing the enhancement of utilization efficiency and the expansion of application potential of existing herbicides represents a more practical and sustainable strategy for managing invasive weeds.

Synthetic herbicides constitute an indispensable cornerstone of agronomic weed management, accounting for approximately 50% of the global pesticide market in recent years [15–17]. Among available active ingredients (AIs), 4-hydroxyphenylpyruvate dioxygenase (HPPD) inhibitors have emerged as a gold-standard owing to their exceptional potency, broad-spectrum bioactivity, and robust crop selectivity [18, 19]. Topramezone (TOP) functions by lethally arresting carotenoid biosynthesis, thereby precipitating catastrophic photooxidative bleaching in weeds [20]. However, the translation of this intrinsic molecular potency into consistent field-level performance is often constrained by environment-dependent bioavailability and environmental dissipation associated with conventional formulations. Current delivery systems exhibit suboptimal utilization efficiency for TOP, and its application is frequently recommended in combination with specific adjuvants [21–24]. These inefficiencies are particularly exacerbated in the control of aggressive invaders like *C. xanthiifolia*, and the necessity for escalating dosages to overcome invasive resilience risks can easily induce non-target phytotoxicity. Consequently, the failure of conventional herbicides to arrest its expansion underscores a critical imperative: the development of precision-targeted and environmentally-integrated interventions for efficient management of invasive *C. xanthiifolia*. Nano-enabled delivery system has emerged as a promising strategy to enhance the efficacy and safety of agrochemicals by improving formulation stability, regulating release behavior, and increasing bioavailability, thereby reducing AI loss and non-target exposure. In contrast, the development of nano-herbicides remains comparatively underexplored, despite their considerable potential to overcome long-standing challenges in weed management. For instance, recent studies have shown that nano-enabled pesticide is predominantly focused on insecticides and fungicides, with herbicide-related studies representing only a minor proportion of reported systems, thereby revealing a significant gap between technological potential and practical application [25, 26]. This imbalance is largely attributable to the physiological complexity and habitat heterogeneity of weeds, which complicate the effective delivery and performance translation at the field level [27, 28].

Amphiphilic hydrophilic–lipophilic diblock polymer (HLDP) nanocarrier has recently emerged as a versatile polymeric delivery platform for agrochemicals. The HLDP can spontaneously self-assemble into hierarchical nanostructures with tunable interfacial properties, enabling the strong interactions between AIs and foliar surfaces. Recent studies have shown that the incorporation of HLDP can improve the foliar wettability, retention and plant uptake of diverse AIs, resulting in superior biological performance of insecticides, fungicides and herbicides [29–32]. Collectively, these features suggest that the HLDP represents a promising nanocarrier to address the intrinsic limitations of HPPD-inhibiting herbicides, including suboptimal utilization and non-target toxicity. Previous HLDP-based herbicide systems (e.g., MP@HLDP) mainly focus on enhanced herbicidal activity, and this study explored the synergistic mechanisms underlying the improved bioactivity at a deeper level. In this context, we developed an HLDP-enabled TOP nano-herbicide and systematically evaluated its control performance against the invasive weed *C. xanthiifolia*. By assessing dynamic self-assembly mechanism and extensive characterization of TOP@HLDP, variation of physiological/molecular responses in weeds, as well as non-target and crop biosafety, our work addressed the key limitations associated with conventional HPPD-inhibiting herbicides for high-efficiency weed management. Our findings demonstrated that the HLDP-assisted delivery could be applied to enhance the effectiveness of herbicides while mitigating potential biological/environmental risks, providing a nano-enabled strategy/rational framework for the design/development of efficient and safe nano-herbicides for invasive weed management.

## Materials and methods

### Chemical reagents

Pure TOP (≥ 97%) and commercial TOP formulation (30% AI) were purchased from Shandong Jingbo Agrochemical Technology Co., Ltd. (China) and BASF (Germany), respectively. The chemical reagents for the synthesis of HLDP contained the ε-caprolactone (ε-CL), 1-butanol, 2-bromo-2-methylpropionyl bromide (BIBB), trimethylamine (TEA, 99%) and Sn(Oct)_2_ bought from Heowns BioChem Technologies (China), tetrahydrofuran (THF), N,N, N′,N′,N″-Pentamethyl diethylenetriamine (PMDETA, 98%) and CuBr (99.999%) bought from Sigma-Aldrich (USA), and 2-(Dimethyl amino) ethyl methacrylate (DMAEMA, 99%) bought from Energy Chemical (China). All other reagents were supplied by Beijing Chemical Works (China). Double distilled water (ddH_2_O) was used throughout all experiments.

### HLDP synthesis

The HLDP, namely poly(ε-CL)-block-poly(2-(dimethylamino)ethyl methacrylate) (PCL-b-PDMAEMA), was synthesized following a previously-reported route [33]. Briefly, linear PCL was prepared via ring-opening polymerization of ε-CL initiated by 1-butanol in the presence of Sn(Oct)₂ at 90 °C for 9 h. The resulting hydroxyl-terminated PCL was converted into the atom-transfer radical polymerization (ATRP) macroinitiator PCL–Br by esterification with BIBB in dry THF with TEA as the base. Subsequently, the PDMAEMA block was grown from PCL–Br via ATRP of DMAEMA using CuBr/PMDETA as the catalytic complex at 65 °C, with a molar ratio of [PCL–Br]: [CuBr]: [PMDETA] ≈ 1:2.5:5.3. After polymerization, the reaction mixture was dialyzed against ddH_2_O to remove unreacted monomers and catalyst residues, followed by lyophilization to obtain the purified copolymer. The chemical structure and block composition were previously verified by ^1^H NMR spectroscopy and gel-permeation chromatography (GPC). Among the obtained copolymers, PCL₄₅-b-PDMAEMA₆₄ was employed for subsequent experiments owing to its well-balanced hydrophobic–hydrophilic ratio.

### Loading capacity measurement of HLDP toward TOP

The loading capacity of HLDP was determined toward TOP using freeze-drying approach. Briefly, 157.5 mg of TOP was dissolved in 20 mL of DMF, followed by adding 2 mL of HLDP aqueous solution (58.3 mg/mL). The mixture was incubated for 15 min to allow complete self-assembly between TOP and HLDP. The resulting suspension was then transferred into regenerated cellulose dialysis bags (MWCO 2000 Da; Shanghai Yuanye Bio-Technology Co., China) and dialyzed for 24 h to remove unassembled TOP. The external dialysis medium was refreshed every 6 h. After dialysis, the retentate was harvested and lyophilized using vacuum freeze-dryer (Beijing Songyuanhuaxing Technology Development Co., China) to obtain the powdered TOP@HLDP. The dried product was weighed, and all experiments were performed in triplicate. The pesticide loading content (PLC) was calculated using the formula of   

$$\rm {PLC\:}\left({\%}\right){\:=\:}\frac{{Weight\:of\:TOP\:loaded\:in\:TOP@HLDP}}{{Weight\:of\:TOP@HLDP}}{\times\:100}$$

### ITC assay and molecular docking/stimulation for the interaction between HLDP and TOP

The interaction between TOP and HLDP was investigated using Nano ITC instrument (TA Instruments, USA). Prior to measurement, all solutions were degassed under vacuum to eliminate dissolved air bubbles. The HLDP aqueous solution (0.138 mM) was loaded into 2 mL sample cell, while the 1 mM TOP solution (250 µL) was placed in injection syringe. Titrations were performed at 25 °C. The raw thermograms were processed using NanoAnalyze software (TA Instruments), and the binding isotherms were fitted to one-site binding model to obtain the thermodynamic parameters, including the binding constant (Ka), enthalpy change (ΔH) and entropy change (ΔS). The Gibbs free energy change (ΔG) was calculated as $\rm {\triangle G\:=\:\triangle H\:}-{\:T\triangle S}$.

Molecular docking was carried out using AutoDock Vina 1.1.2 (The Scripps Research Institute, USA) to characterize the interaction mode between TOP and HLDP [34]. The molecular structures of TOP and HLDP were constructed in ChemDraw Professional (PerkinElmer, USA), converted to 3D conformations in Chem3D, and fully optimized using Gaussian 16 (Gaussian Inc., USA). Restricted electrostatic potential (RESP) charges were calculated from the optimized structures using Multiwfn 3.8, and force-field parameters were produced under GAFF using Sobtop 2.1. Docking poses were scored using Vina scoring function, and intermolecular interactions were observed in PyMOL 3.7 (Schrödinger, USA) [35]. To further investigate dynamic self-assembly behavior and binding stability of TOP@HLDP in aqueous solution, all-atom molecular dynamics (MD) simulations were carried out using GROMACS 2024.3 [36]. A cubic box (5 × 5 × 5 nm³) containing 10 molecules of HLDP and TOP was solvated with TIP3P water, followed by energy minimization using steepest-descent algorithm [37]. The system was equilibrated under NVT and NPT ensembles (300 K, 1 bar), and a 100 ns production run was conducted. PME was applied for long-range electrostatics, and all covalent bonds were constrained with LINCS [38, 39]. The trajectories were analyzed to evaluate conformational stability, compactness and intermolecular interactions.

### Extensive characterization analysis of TOP@HLDP

For physicochemical characterization, the morphology, microstructure, hydrodynamic diameter, polydispersity, zeta potential, Fourier transform infrared spectroscopy (FTIR), colloidal stability and thermal behavior of TOP@HLDP were extensively examined in the current study. In addition, the hydrodynamic diameter and zeta potential of the commercial TOP were measured for comparison. The TOP@HLDP was prepared as follows in all subsequent experiments unless otherwise stated. The TOP was dissolved in DMF, and then added dropwise into the HLDP aqueous solution under gentle stirring, ensuring that the final DMF content did not exceed 1% (v/v). For spontaneous self-assembly of TOP@HLDP, two components were incubated at room temperature for 15 min at the optimal mass ratio of 1:1.16 (TOP: HLDP) according to the PLC.

The morphology and microstructure of TOP@HLDP (mass ratio of 1:1.16; TOP concentration: 1 mg/mL) and TOP alone were firstly examined by transmission electron microscopy (TEM, Hitachi HT7700, Japan) and scanning electron microscopy (SEM, JSM-6700 F, Japan). For TEM observation, a droplet of diluted sample was deposited onto a carbon-coated copper grid and air-dried at room temperature prior to imaging. Energy-dispersive X-ray spectroscopy (EDS) was further conducted during TEM observation to determine the elemental composition of samples. The characteristic signals of Br (derived from HLDP) and S (derived from TOP) were used as fingerprints to verify the formation of TOP@HLDP. For SEM observation, single-crystal silicon wafers (5 × 5 mm) were fixed onto sample stubs using double-sided carbon conductive tape. A 20 µL of each sample was deposited onto the silicon wafer surface and air-dried, which was then sputter-coated with platinum and observed. Furthermore, the hydrodynamic diameter, polydispersity and zeta potential of TOP@HLDP (mass ratios of 1:1 and 1:1.16; TOP concentration: 1 mg/mL) and TOP alone were determined using dynamic light scattering (DLS) instrument equipped with a zeta potential analyzer (NanoBrook Omni, Brookhaven Instruments, USA). Each sample was freshly prepared and tested for three times.

The FTIR was used to analyze the functional groups and intermolecular interactions of TOP@HLDP, TOP and HLDP using IRTracer-100 spectrometer (Shimadzu, Japan). All samples were examined in solid form using KBr pellet method. FTIR spectra were recorded over 4000–500 cm⁻¹ at a resolution of 4 cm⁻¹, and each spectrum represented the average of 32 scans.

To assess the colloidal stability, the particle size and zeta potential of TOP@HLDP (mass ratios of 1:1.16; TOP concentration: 1 mg/mL) were monitored at different temperatures (5, 15, 25, 35 and 45 °C). Storage stability was also evaluated by determining the same parameters after 1, 3, 5, 7 and 9 d of storage. All measurements were performed in triplicate. In addition, the thermal behavior of TOP@HLDP, TOP and HLDP was investigated using simultaneous thermogravimetric–differential scanning calorimetry system (TGA/DSC 3+, Mettler Toledo, Switzerland). Approximately 10 mg of each sample was placed in an alumina crucible and heated from 30 to 600 °C at a rate of 10 °C/min under nitrogen to prevent oxidative degradation. Thermogravimetric (TG) and DSC curves were constructed throughout the heating process.

### Foliar deposition and uptake behavior test of TOP@HLDP

The foliar behavior of TOP@HLDP was evaluated and compared with free/commercial TOP by examining leaf wettability, spray retention and systemic uptake. The tested solutions of TOP@HLDP (mass ratios of 1:1.16), free TOP and commercial TOP were prepared at a field-relevant TOP concentration of 60 mg/L. The corresponding amount of HLDP in the TOP@HLDP formulation was determined according to the predefined mass ratio. The HLDP-only solution was prepared at the same HLDP concentration in the TOP@HLDP formulation. Leaf wettability was assessed by measuring the static contact angle of droplets on the adaxial surface of *C. xanthiifolia* leaves using contact angle goniometer (OCA25, DataPhysics Instruments, Germany). A 5 µL droplet of each solution was gently deposited on the leaf surface, and the contact angle was recorded after 10 s. Ten biological replicates were analyzed for each solution. For retention test, 1-naphthyl isothiocyanate (FITC) was added into each solution to yield a final FITC concentration of 0.2 mg/mL for fluorescence detection. The discs of *C. xanthiifolia* leaves (surface area: 0.785 cm²) were harvested. The initial weight of each disc was recorded using an analytical balance (accuracy: 0.1 mg), followed by immersion in the test solution for 30 s. The discs were then suspended vertically until dripping ceased, after which the final weight was measured. Retention was calculated from eight independent replicates using the following formula:

$$\begin{aligned}&\rm {Retention\:(mg/}{{cm}}^{{2}})= \\& \quad\rm \frac{{Disc\:mass\:after\:immersion\:}-{\:Disc\:mass\:before\:immersion}}{{Leaf\:surface\:area}} \end{aligned}$$

To evaluate the systemic uptake of HLDP-loaded TOP, intact *C. xanthiifolia* seedlings were exposed to TOP@HLDP (mass ratios of 1:1.16; TOP concentration: 1 mg/mL) and free TOP via root immersion, respectively. At 3, 6 and 9 h after treatment, 0.1 g of fresh leaf tissue was harvested from each seedling, immediately frozen in liquid nitrogen, and ground thoroughly. The powdered samples were extracted with 1.8 mL acetonitrile/water (10:1, v/v), followed by centrifugation. Then, 1.5 mL of the supernatant was transferred into a clean tube, added with 0.054 g NaCl, vortexed for 10 min, and allowed to stand for 5 min to facilitate phase separation. The upper organic phase (1.2 mL) was harvested, sonicated, and filtered through a 0.22 μm membrane filter prior to quantitative analysis. HPLC analysis was carried out using a reversed-phase C18 column. The mobile phase consisted of acetonitrile/water (75:25, v/v) delivered at a flow rate of 0.30 mL/min, with the column temperature maintained at 30 °C. The detection wavelength was set at 280 nm. All experiments were conducted in triplicate. A calibration curve for TOP was constructed using standard solutions ranging from 0.001 to 1 mg/mL, which was used to quantify the TOP.

The *C. xanthiifolia* seedlings were treated with TOP or TOP@HLDP (mass ratios of 1:1.16; TOP concentration: 60 mg/L) via root application for 2 d, and the stems were excised and dissected into small fragments (approximately 1–2 mm³). The samples were fixed in Fine Active Alumina solution, followed by paraffin embedding. Transverse sections with a thickness of 5 μm were prepared using rotary microtome and mounted on glass slides. To visualize tissue structures, the sections were sequentially stained with safranin O (0.1% w/v) and fast green FCF (0.05% w/v) (CAB-30PC, Cabontek Co., China). Hyperspectral imaging (HSI) was performed using CytoViva enhanced dark-field hyperspectral imaging (EDF-HSI) system (CytoViva, Inc., USA), equipped with Olympus BX43 dark-field microscope (Olympus Corporation, Japan), 150 W halogen light source, and CytoViva hyperspectral camera with CCD detector. Dark-field images of stem sections were acquired at 60× magnification under oil immersion (numerical aperture = 1.25). Hyperspectral data were obtained using ENVI software (Exelis Visual Information Systems, Boulder, CO., USA). The illumination intensity was set to 100%, and both acquisition and exposure times were fixed at 0.1 ms. Each pixel in the hyperspectral images contained continuous spectral information spanning the wavelength range of 400–1000 nm, with a spectral resolution of 3 nm.

### Soil leaching analysis of TOP@HLDP

The vertical mobility of TOP@HLDP and free TOP was assessed in soil using soil column leaching assay. Field topsoil was harvested, air-dried, and sieved through a 0.2-mm mesh to obtain a homogeneous matrix. Soil columns were prepared using 50-mL centrifuge tubes perforated at the base to allow leachate drainage. A cotton layer (5 cm^3^) was placed at the bottom of each tube, followed by adding 40 cm^3^ of sieved soil. A 5 cm^3^ layer of silica sand (Tianjin Kemiou Chemical Reagent Co., China) was added to the top, and the column surface was covered with filter paper. Prior to sample application, the soil columns were pre-wetted with 0.01 mol/L CaCl₂ solution prepared from anhydrous CaCl₂ (Sinopharm Chemical Reagent Co., China) to simulate field ionic strength and ensure uniform moisture distribution. Free TOP or TOP@HLDP (mass ratio of 1:1.16; 10 mg TOP per column) was then applied to the soil surface. The columns were subsequently eluted with ddH_2_O, and the leachate was harvested in 5-mL fractions, yielding ten sequential eluates per column. The concentration of TOP in each fraction was determined by HPLC similarly as above, using an external calibration curve (y = 20993x). The analytical sensitivity was evaluated based on low-concentration responses (*n* = 3), and the limit of detection (LOD) and limit of quantification (LOQ) were estimated using LOD = 3.3σ/S and LOQ = 10σ/S, where σ is the standard deviation of the response and S is the slope of the calibration curve. All treatments were performed in triplicate, and the residual TOP was calculated using the following formula:

$$\rm Residual \,TOP (\%) = (N1 - N2)\div N1\times100$$

N1 is the total amount of TOP, and N2 is the amount of TOP eluted from the soil column.

### Herbicidal activity assay of TOP@HLDP in the laboratory

The herbicidal activity of TOP@HLDP and free TOP was assessed using *C. xanthiifolia* seedlings at the four-leaf growth stage. Four treatments included TOP@HLDP (mass ratio of 1:1.16), free TOP, HLDP and ddH_2_O (control). For the treatments with TOP@HLDP and TOP, three TOP concentrations (3.75, 7.5 and 15 mg/L) were prepared. The test solutions were applied uniformly to the foliage using hand sprayer, with 1.0 mL solution per seedling. Following application, the seedlings were maintained under controlled conditions (26 ± 2 °C, 16 h light/8 h dark photoperiod). Plant height was recorded at 0, 2, 4, 6, 8, 10, 12 and 14 d after the treatment. The plant height increment at each time point was calculated as the difference between the height at day t (H_t_) and the initial height at day 0 (H_0_). Each treatment included eight independent seedlings. Furthermore, the leaf physiological traits, such as chlorophyll index, nitrogen index and leaf surface moisture, were measured using portable chlorophyll meter (Hangzhou Greenbio Instrument Co., China). Measurements were performed from the middle region of the 3rd fully-expanded leaves, and three readings per plant were averaged. Each treatment contained eight independent seedlings.

### Herbicidal activity assay of TOP@HLDP in the field

The field performance of TOP@HLDP was evaluated against *C. xanthiifolia* seedlings under natural field conditions. Five treatments included TOP@HLDP (mass ratio of 1:1.16; TOP concentration: 15 mg/L), free TOP (TOP concentration: 15 mg/L), commercial TOP formulation (TOP concentration: 30 mg/L), HLDP and ddH_2_O (control). Seedlings at the four-leaf stage were transplanted with approximately thirty plants per square meter. All test solutions were applied foliarly using hand-held sprayer at a dosage of 45 mL/m^2^. Plant height was recorded at 0, 2, 4 and 7 d after the treatment. The height increment at each time point was calculated as the difference between the height at day t (H_t_) and the baseline value at day 0 (H_0_). Herbicidal activities of above solutions were evaluated at 7 d after the treatment via the visual method. Injury was estimated as a percentage (0–100%) based on the extent of chlorosis, bleaching, and necrosis observed in the seedlings, where 0% represents no visible damage and 100% represents complete plant death. For guidance, the following reference criteria were used: 0 = no damage or normal growth; 0–30% = minority whitening of leaves; 30%-60% = newborn leaves showed whitening; 60%-100% = majority of seedling parts showed whitening and necrosis; 100% = whole seedling showed whitening and necrosis. In addition, the above-ground tissues were harvested at 7 d after the treatment for biomass determination. Fresh weight was measured immediately after collection, and dry weight was determined following oven-drying at 80 °C until constant mass. Fresh weight control efficacy (%) was calculated using the equation of Fresh weight control efficacy (%) = (1-FW_treatment_ ÷ FW_control_) × 100, where FW_treatment_ and FW_control_ represent the mean fresh biomass of each treatment and ddH_2_O group, respectively. All above treatments included ten independent seedlings. The leaf physiological parameters, including chlorophyll index, nitrogen index and leaf surface moisture were also recorded similarly as above. Three readings per plant were averaged, and each treatment contained ten independent seedlings.

### Transcriptomic and metabolomic analysis for the synergistic mechanism of HLDP-loaded TOP

The *C. xanthiifolia* seedlings were treated with TOP@HLDP and free TOP similarly to the above operations in the laboratory, and the leaf tissues were harvested at 8 d after the foliar application. For transcriptomic analysis, each treatment consisted of three independent biological replicates, while six biological replicates were used for metabolomic analysis.

For transcriptome analysis, total RNA was extracted using TRIzol reagent (TIANGEN, China) according to the manufacturer’s protocol. RNA yield, purity and integrity were assessed prior to library preparation. Sequencing libraries were constructed and sequenced on Illumina high-throughput platform. Raw reads were trimmed to remove adaptor sequences and low-quality reads (reads containing > 50% bases with Q ≤ 10), and the resulting high-quality clean reads were used for downstream analyses. As no reference genome is currently available for *C. xanthiifolia*, de novo transcriptome assembly was performed using Trinity [40]. The assembled unigenes were functionally annotated by BLAST alignment against multiple public databases. Gene expression levels were normalized as fragments per kilobase of transcript per million mapped reads (FPKM). Differential expression analysis between TOP@HLDP and TOP treatments was conducted using DESeq2, with screening thresholds set at |fold change| ≥ 1 and *P* < 0.1 [41]. Identified differentially expressed genes (DEGs) were subsequently subjected to functional classification and KEGG pathway enrichment analysis.

For metabolomic analysis, approximately 100 mg of frozen leaf tissue was extracted using pre-chilled methanol/acetonitrile/water (2:2:1, v/v/v). After vortex mixing, low-temperature ultrasonication and centrifugation, the supernatant was dried under vacuum and re-dissolved in acetonitrile/water (1:1, v/v) for instrumental analysis. Untargeted metabolomic profiling was performed using ultra-high-performance liquid chromatography system (Agilent 1290 Infinity LC, USA) coupled to high-resolution mass spectrometer (TripleTOF 6600, AB SCIEX, USA) equipped with electrospray ionization source operating in both positive and negative modes. Chromatographic separation was achieved using an ACQUITY UPLC BEH Amide column under HILIC conditions. Quality control (QC) samples were injected regularly throughout the analytical sequence to assess system stability. Raw data were converted and processed for peak detection, alignment and normalization using XCMS. Putative metabolite annotation was achieved based on accurate mass, retention time and MS/MS spectra by matching against public databases [42]. Multivariate analyses, including principal component analysis (PCA) and partial least-squares discriminant analysis (PLS-DA), were performed to evaluate metabolic divergence between treatments. Differential metabolites were defined using a fold-change cutoff of ≥ 1 and *P* < 0.1, which were subsequently subjected to KEGG pathway enrichment analysis. The samples were subjected to a combined analysis of transcriptomics and metabolomics.

### Quantification of key genes and biochemical indicators in *C. xanthiifolia* treated with TOP@HLDP

Above RNA-seq samples were used to synthesize first-strand cDNA using PrimeScript RT Kit (Takara, Japan). Quantitative real-time PCR (qRT-PCR) was performed on ABI QuantStudio 6 Flex system (Thermo Fisher Scientific, USA) using TB Green Premix Ex Taq II (Takara). The thermal cycling program consisted of 95 °C for 30 s, followed by 40 cycles of 95 °C for 5 s and 60 °C for 34 s. Relative transcript abundance was calculated using the 2^⁻ΔΔCt^ method, with *EF1α* serving as the internal reference gene [43]. Primer sequences are shown in Table S1.

The *C. xanthiifolia* seedlings were treated with TOP@HLDP and TOP similarly to the above operations in the laboratory. The leaf tissues (0.1 g) were harvested at 1 d after the foliar application, and immediately frozen in liquid nitrogen to quantify superoxide anion (O₂⁻), hydrogen peroxide (H₂O₂), malondialdehyde (MDA), peroxidase (POD), superoxide dismutase (SOD), catalase (CAT), glutathione S-transferase (GST), glutathione (GSH) and tyrosine using corresponding assay kits supplied by Beijing Solarbio Science & Technology Co., Ltd. (China). The leaf tissues of above treated seedlings were also harvested at 24, 72 and 144 h after the foliar application, which were employed to quantify carotenoid content using commercial assay kit (Shanghai Yuanye Bio-Technology Co., Ltd., China). All measurements were conducted using three independent biological replicates.

### Biosafety assessment of TOP@HLDP toward fruit fly, S2 cell and maize seed

The security evaluation of TOP@HLDP toward terrestrial insects was assessed using oral exposure assay with adults of fruit flies (*Drosophila melanogaster*, Canton-S strain). The fly adults were fed with 4% sucrose containing TOP@HLDP (mass ratio of 1:1.16; TOP concentration: 12, 15 and 18 mg/L), free TOP, HLDP or ddH_2_O. The flies were maintained at 26 ± 1 °C and 60–70% relative humidity, and their survival rate was recorded at 24, 48, 72 and 96 h post-exposure. Each treatment contained twenty adults, which was repeated three times.

To further examine biosafety at the cellular level, *Drosophila* S2 cells were cultured in Schneider’s *Drosophila* medium (Gibco, USA) supplemented with 10% fetal bovine serum at 28 °C, which were then seeded into 96-well plates at a density of 1 × 10⁴ cells per well, and exposed to TOP@HLDP (mass ratio of 1:1.16; TOP concentration: 6, 9, 12, 15, 18, 21 and 24 mg/L), free TOP or ddH_2_O (control) for 24 h. For alive/dead cell discrimination, cells were further stained using Calcein-AM/propidium iodide (PI) dual-fluorescence viability kit according to the manufacturer’s instructions. Alive cells exhibited green fluorescence, whereas membrane-compromised dead cells were stained by red. Fluorescence images were captured using inverted fluorescence microscope (AMG, USA). Cell viability was determined using CCK-8 assay (Solarbio, China), and absorbance was measured at 450 nm using microplate reader (Thermo Fisher Scientific, USA). Relative cell viability was calculated using five biological replicates against the control (ddH_2_O) according to the following equation:

$$\begin{aligned}& \rm Cell viability (\%) = \\& \rm(Absorbance of treatment - Absorbance of blank) \\& \div \rm(Absorbance of control - Absorbance of blank) \\&\times 100\end{aligned}$$

The biosafety of TOP@HLDP toward crop plants was evaluated using maize (*Zea mays* L.) seeds. Uniform and healthy seeds were randomly divided into three treatment groups: ddH_2_O (control), free TOP and TOP@HLDP (mass ratio of 1:1.16; TOP concentration: 15 mg/L). Seeds were cultured on the filter paper containing 2 mL above solution at 25 °C under consistent humidity conditions with normal light exposure. Germination and seedling growth were evaluated at 5 d after treatment. Seeds were considered germinated when the radicle had clearly emerged from the seed coat and reached a length of at least 2 mm. Germination rate (%) was calculated as the percentage of germinated seeds relative to the total number of seeds. In addition, primary root length was measured as an indicator for seedling growth. Root length was defined as the distance from the radicle emergence point at the seed coat to root tip. Each treatment consisted of three biological replicates with ten seeds per replicate.

### Statistical analysis

Statistical analyses were performed using IBM SPSS Statistics 26.0 (IBM Corp., USA). Significant differences between two groups were evaluated using independent *t*-test. For comparisons among multiple treatments, one-way analysis of variance (ANOVA) was conducted, followed by Tukey’s honestly significant difference (HSD) post hoc test. A significance threshold of *P* < 0.05 was adopted for all analyses. All quantitative data are expressed as mean ± standard error (SE).

## Results and discussion

### High loading capacity of HLDP toward TOP

The loading performance of HLDP was evaluated after dialysis to remove unincorporated TOP. Based on quantitative analysis of the retained TOP, the PLC of HLDP was determined to be 46.22% (Table 1). This loading level falls within the typical range previously reported for HLDP-based delivery systems, with the PLC values of 16.51% for monosultap (Mon), 21.71% for metamifop (MP), 48.49% for salicylic acid (SA) and 51.21% for chitosan [29, 30, 32, 44]. The capacity of HLDP to encapsulate chemically-diverse AIs can be attributed to its amphiphilic block-copolymer structure, which combines hydrophobic PCL segments with hydrophilic PDMAEMA chains, thereby enabling multiple non-covalent interactions during the self-assembly. As a consequence, HLDP readily forms stable nanocomposites with a broad spectrum of small-molecule agrochemicals. Such loading behavior highlights the versatility of HLDP as a nanocarrier/adjuvant and supports its potential application in developing advanced pesticide formulations.

**Table 1:** Loading capacity of HLDP toward TOP using freeze drying method

Sample number	Weight of applied TOP (mg)	Weight of applied HLDP (mg)	Weight of TOP@HLDP (mg)	Weight of TOP loaded in TOP@HLDP (mg)	Drug-loading content (%)	Average drug-loading content (%)
1	157.5	116.6	220.0	103.4	47.00	46.22 ± 0.40
2	157.5	116.6	214.8	98.2	45.72	
3	157.5	116.6	215.7	99.1	45.94	

Mean ± SE

### Dynamic self-assembly of TOP@HLDP via multiple interaction forces

**Fig. 1:**
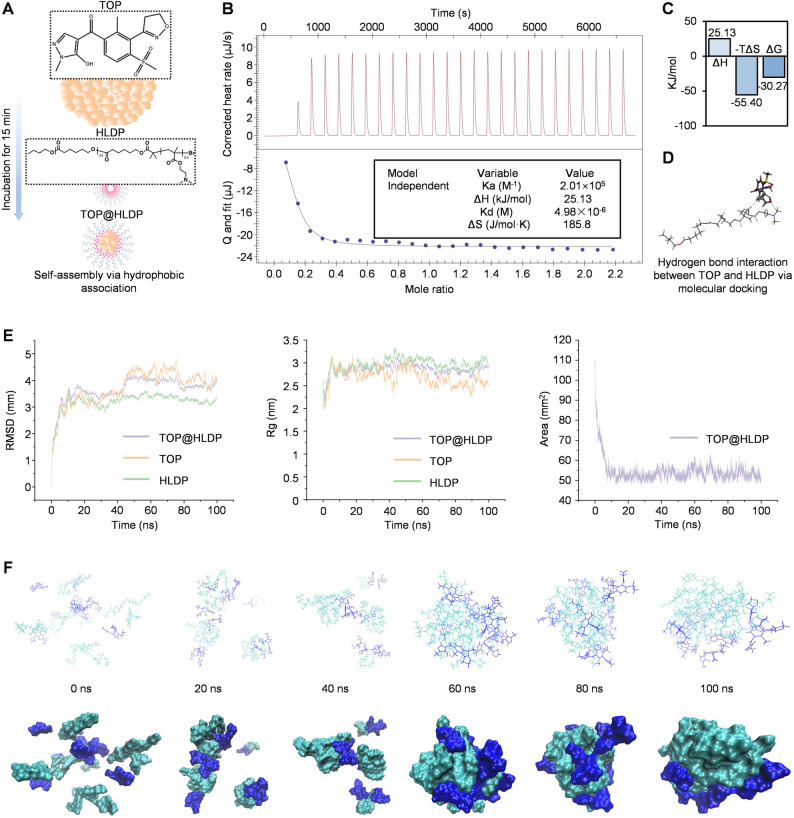
Self-assembly mechanism and molecular interaction between TOP and HLDP. (**A**) Schematic illustration of the preparation route for TOP@HLDP. (**B**,** C**) ITC profiles obtained by titrating TOP solution (1 mmol/L) into HLDP (0.138 mmol/L) solution. (**D**) Interaction between TOP and HLDP at 100 ns. (**E**) MD simulation analysis of the TOP@HLDP, including the time evolution of RMSD, Rg and SASA. (**F**) Representative snapshots from 0 to 100 ns MD simulations, illustrating dynamic self-assembly process of TOP (blue) within HLDP (green). TOP and HLDP are represented using stick models

The ITC was firstly conducted to investigate the driving forces for the self-assembly of TOP@HLDP. The spontaneous binding process was characterized by a negative ΔG (-30.27 KJ/mol), and the thermodynamic profile displayed a pronounced positive ΔH (25.13 kJ/mol) together with a positive ΔS (185.8 J/mol·K) (Fig. 1A-C). Such an enthalpy–entropy compensation pattern is typically indicative of hydrophobic association, in which entropy gain mainly originates from the release of ordered interfacial water molecules from non-polar surfaces during complex formation. This thermodynamic signature is in good agreement with the molecular structure of TOP that contains both substituted benzene ring and N-methyl-pyrazole moiety, providing extended hydrophobic surfaces capable of partitioning into the lipophilic domains of HLDP. These results are also consistent with a previous report showing that HLDP can associate with chemically-diverse AIs through multiple non-covalent forces, including electrostatic interactions with SA, hydrogen bonding/Van der Waals forces with carbendazim, and hydrophobic interactions with prochloraz [29, 30].

To further elucidate the molecular interaction patterns, molecular docking analysis was conducted. The results showed that the TOP was preferentially assembled within HLDP via hydrogen bonds (Fig. 1D). All-atom molecular dynamics (MD) simulations were subsequently performed for 100 ns to characterize the dynamic stability and self-assembly behavior of TOP@HLDP (Fig. 1E-F). The RMSD trajectory displayed an initial rise during the first 10 ns and then reached a stable plateau, suggesting that the TOP@HLDP underwent a short conformational adaptation phase prior to achieving equilibrium [45, 46]. Meanwhile, the radius of gyration (Rg) gradually increased and stabilized at 40 ns, indicating enhanced structural compactness of TOP@HLDP over time [47, 48]. A similar trend was observed for the solvent-accessible surface area (SASA), which declined markedly during the early stage and then stabilized at 10 ns, consistent with the progressive burial of hydrophobic surfaces and reduced solvent exposure as the nanocomplex condensed into a compact architecture [49, 50]. Representative structural snapshots showed a clear evolution from initially-dispersed molecules (0 ns) to compact nanostructures at later stages, consistent with the convergent trends in RMSD, Rg and SASA (Fig. 1F). Our findings together demonstrated that hydrogen bonding provided additional stabilization to collectively support the formation of structurally-robust TOP@HLDP in aqueous solution, while hydrophobic association constituted the dominant driving force for the self-assembly of TOP@HLDP.

### Reduced particle size and enhanced stability of HLDP-loaded TOP

**Fig. 2:**
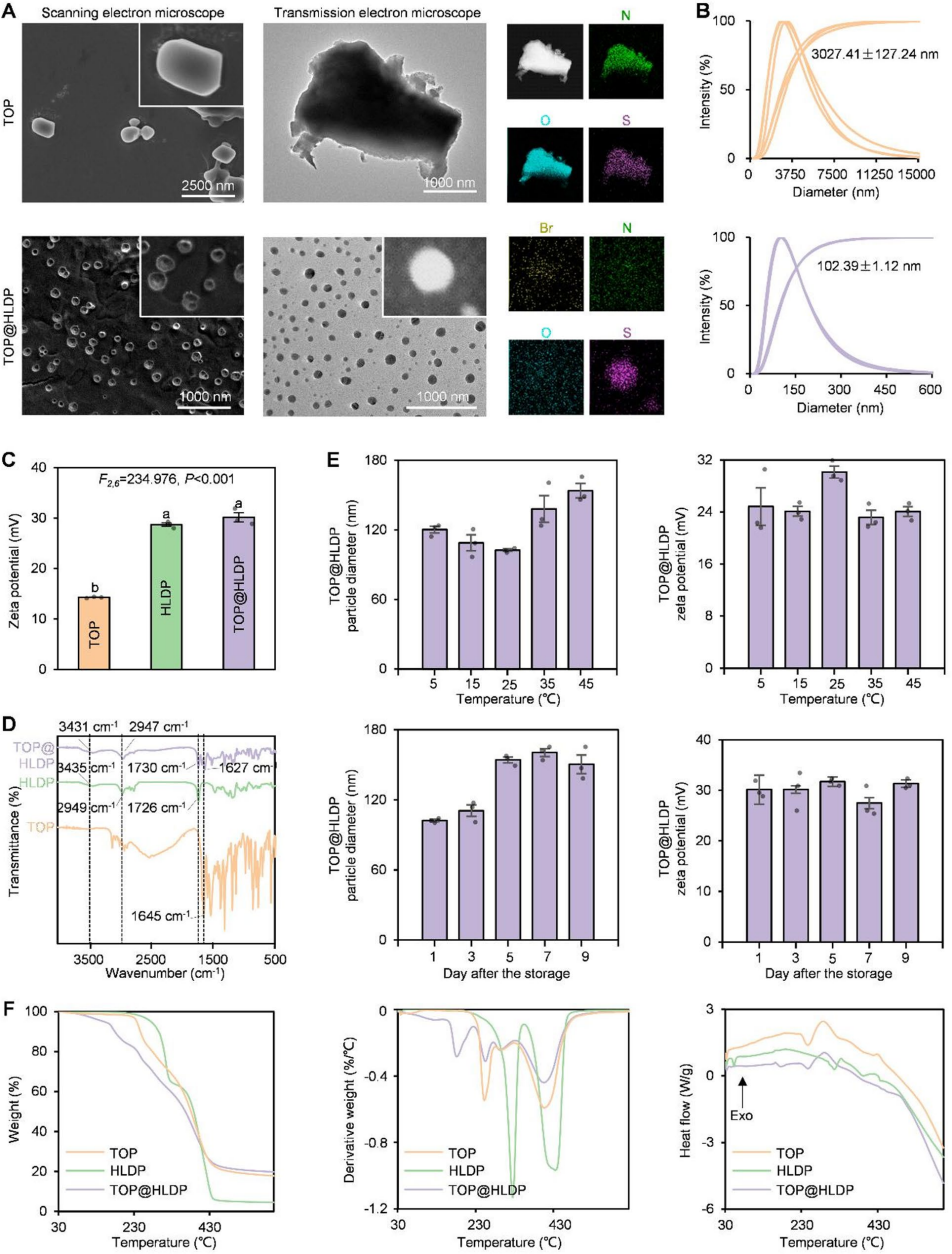
Characterization of TOP@HLDP. (**A**) SEM and TEM images of TOP and TOP@HLDP, together with corresponding elemental mapping. (**B**) Particle size distributions of TOP and TOP@HLDP. Each treatment included three independent samples. (**C**) Zeta potentials of TOP, HLDP and TOP@HLDP. Each treatment included three independent samples. Different letters above each bar indicate significant difference at *P* < 0.05 as determined by one-way ANOVA with Tukey HSD test. (**D**) FTIR spectra of TOP, HLDP and TOP@HLDP. (**E**) Particle size and zeta potential of TOP@HLDP under different temperatures (5–45 °C) and during 1–9 d storage. Each treatment included three independent samples. (**F**) TG-DSC curves of TOP, HLDP and TOP@HLDP

The morphology and microstructure of TOP@HLDP were examined by SEM and TEM (Fig. 2A). Free TOP appeared as irregular, block-like crystalline aggregates, whereas TOP@HLDP formed predominantly spherical or near-spherical nanoparticles with smooth surfaces and uniform morphology. This transformation from bulk crystals to discrete nanostructures indicated effective encapsulation of TOP within the amphiphilic matrix of HLDP. EDS elemental mapping further confirmed the hybrid nature of the TOP@HLDP: both characteristic sulfur (S) signals from the methylsulfonyl group of TOP and bromine (Br) signals from the quaternary ammonium terminus of HLDP were homogeneously distributed throughout the particles, providing the direct evidence that both components were incorporated into the same nanostructured entities. Furthermore, the DLS results revealed a dramatic reduction in particle size of TOP upon complexation. Free TOP rapidly formed micron-scale aggregates in aqueous suspension (3027.41 nm), whereas the TOP@HLDP exhibited a mean hydrodynamic diameter of 102.39 nm with a narrow size distribution (PDI = 0.234) at the mass ratio of 1:1.16 (Table 2; Fig. 2B). The zeta potential of TOP@HLDP was + 30.14 mV, reflecting the influence of the cationic quaternary-ammonium end groups in HLDP (Fig. 2C). For comparison, the particle size distribution and zeta potential of the commercial TOP were also determined, and the corresponding results are presented in Fig. S1A-B. This nanoscale dispersion of TOP@HLDP is consistent with colloidal behavior reported for other HLDP-based delivery systems, and the complexation with HLDP can enhance the stability and dispersion of SA and MP [30, 32].

**Table 2:** Polydispersities and particle sizes of TOP and TOP@HLDP

Formulation	Mass ratio	Sample number	Polydispersity	Average polydispersity	Size (nm)	Average size (nm)
TOP	-	1	0.279	0.374 ± 0.057 a	3261.40	3027.41 ± 127.24 a
		2	0.475		2997.03	
		3	0.367		2823.79	
TOP@HLDP	1:1	1	0.346	0.378 ± 0.020 a	154.67	159.16 ± 3.05 b
		2	0.374		157.83	
		3	0.414		164.97	
	1:1.16	1	0.221	0.234 ± 0.029 a	104.32	102.39 ± 1.12 b
		2	0.254		102.41	
		3	0.228		100.44	
			*F*_*2,6*_ = 5.411, *P* = 0.045		*F*_*2,6*_ = 518.088, *P* < 0.001	

The Tukey’s HSD test was used to analyze the data at *P* = 0.05 level of significance

The FTIR spectroscopy was further applied to elucidate the intermolecular interaction within the composite system (Fig. 2D). Free TOP exhibited characteristic absorption band at 1645 cm⁻¹ attributable to vibrations associated with the heteroaromatic moieties of the molecule. In contrast, HLDP displayed a dominant ester carbonyl stretching band at 1726 cm⁻¹ together with aliphatic C–H stretching near 2947 cm⁻¹, but no absorption feature was observed around 1645 –1627 cm⁻¹. Interestingly, the principal bands derived from both components were retained in the spectrum of TOP@HLDP. Notably, in addition to the ester carbonyl band at 1730 cm⁻¹, a well-defined absorption feature appeared at approximately 1627 cm⁻¹, which was absent in HLDP and slightly down-shifted relative to the 1645 cm⁻¹ band of free TOP. Meanwhile, HLDP also displayed a broad band at 3435 cm⁻¹ assignable to O–H/N–H vibrations, which shifted slightly to 3431 cm⁻¹ in the TOP@HLDP. The emergence of the 1627 and 3431 cm⁻¹ bands in TOP@HLDP indicated that the incorporation of TOP into HLDP led to microenvironmental rearrangement and weak non-covalent interactions, most plausibly involving hydrogen bonding and hydrophobic packing within the polymer domains.

The stability of TOP@HLDP was evaluated under application-relevant conditions by monitoring hydrodynamic diameter and zeta potential across temperatures from 5 to 45 °C and storage intervals from 1 to 9 d (Fig. 2E). The zeta potential remained essentially unchanged across all tested conditions, indicating that the surface physicochemical properties were maintained. Although the particle size increased slightly with both higher temperature and longer storage (from 102 nm at 25 °C to 154 nm at 45 °C and from 102 to 160 nm after 7 d storage), the particles remained within the nanoscale range without macroscopic aggregation, precipitation or phase separation. Such robustness supported the conclusion that the HLDP effectively prevented re-crystallization and aggregation of TOP. Meanwhile, the TG–DSC analysis was performed to reveal distinct thermal behaviors for TOP, HLDP and TOP@HLDP (Fig. 2F). Free TOP exhibited a single major mass-loss event beginning at approximately 230 °C and completing near 430 °C, consistent with the thermal decomposition of the crystalline AIs. In contrast, the HLDP showed two discrete decomposition stages, with sharp DTG peaks indicating a stepwise structural collapse. Notably, TOP@HLDP displayed a broadened and slightly-shifted mass-loss profile relative to both components, together with attenuated DTG peak intensity, suggesting a more gradual degradation process. This indicated that the TOP was molecularly dispersed within HLDP matrix rather than simply mixed in a physical blend. DSC thermograms further supported this interaction: TOP exhibited pronounced exothermic transitions, whereas HLDP showed weaker thermal events; however, the TOP@HLDP displayed dampened heat-flow responses and reduced transition intensity. The suppressed exothermicity and delayed thermal degradation collectively demonstrated that the complexation with HLDP enhanced the thermal stability of TOP by forming a hybrid nanostructure that restricted molecular mobility and retarded volatilization and decomposition. Therefore, above physicochemical characterizations confirmed the successful formation of TOP@HLDP with well-defined nano-morphology, homogeneous elemental distribution, excellent colloidal stability, and strong thermal resistance.

### Stronger foliar adhesion, plant uptake and soil migration of HLDP-loaded TOP

**Fig. 3:**
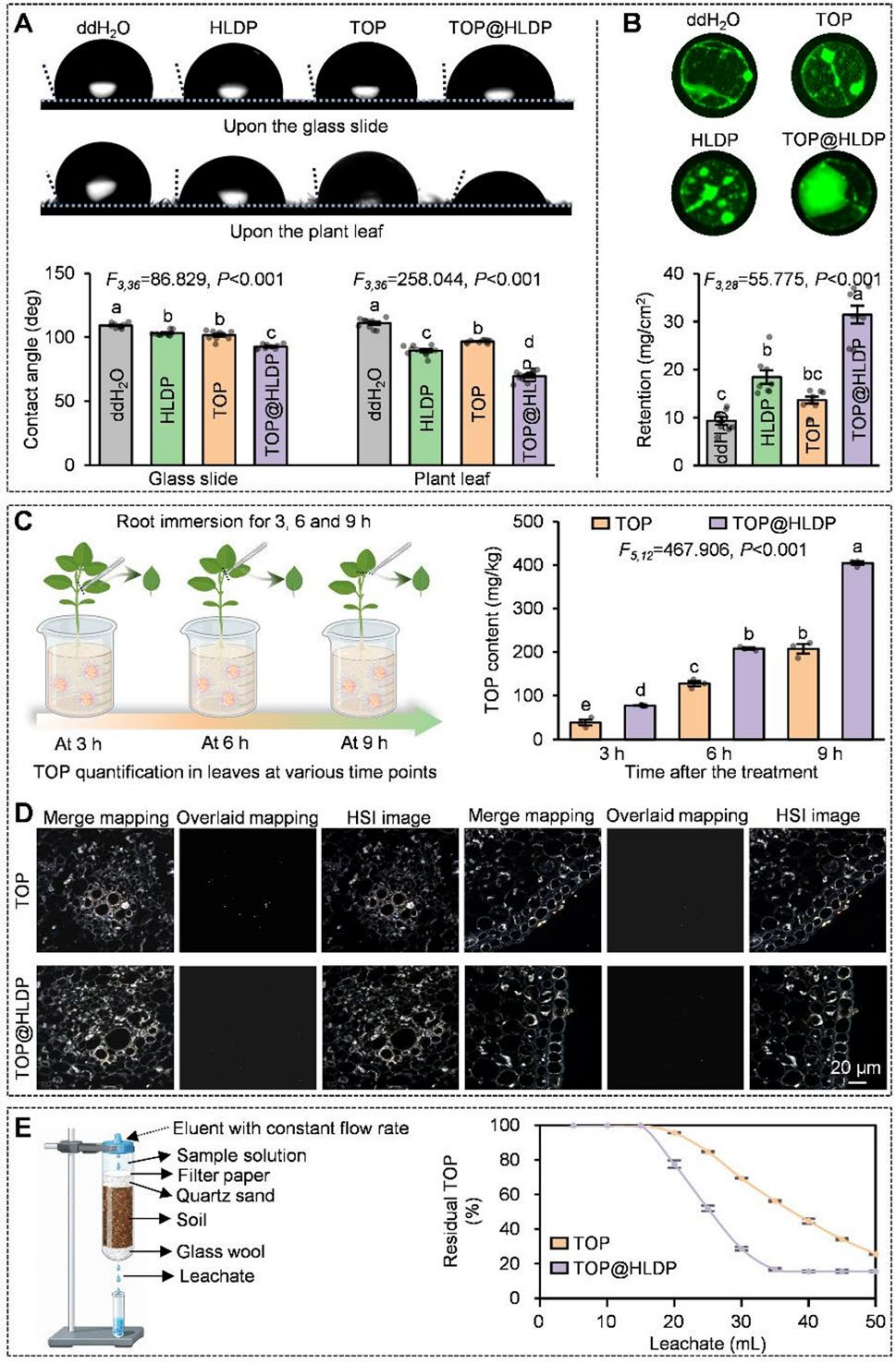
Plant uptake behavior and soil mobility of TOP and TOP@HLDP. (**A**) Contact angles of TOP and TOP@HLDP (60 mg/L) on the leaf surface of *C. xanthiifolia*. Each formulation was tested ten times. Different letters above each bar indicate significant difference at *P* < 0.05 as determined by one-way ANOVA with Tukey HSD test. (**B**) Retention of TOP and TOP@HLDP on the leaf surface of *C. xanthiifolia*. Each treatment was repeated eight times. (**C**) Uptake of TOP@HLDP by *C. xanthiifolia*. Each treatment was repeated three times. (**D**) Representative hyperspectral images of stem tissues from *C. xanthiifolia* treated with TOP and TOP@HLDP. (**E**) Residual TOP in soil after the treatment with TOP and TOP @HLDP. Each treatment was repeated three times

Efficient droplet deposition and subsequent uptake are key determinants of utilization efficiency for herbicides under field conditions. Therefore, the wetting and adhesion behaviors of TOP@HLDP on *C. xanthiifolia* leaves were firstly assessed by measuring static contact angle. As shown in Fig. 3A, TOP exhibited a relatively large contact angle on the leaf surface (96.76°), consistent with the intrinsic hydrophobicity of both TOP and plant cuticle. In contrast, the complexation with HLDP markedly decreased its contact angle to 69.68°, indicating significantly better spreading of droplets. For comparison, the contact angle of the commercial TOP under the same conditions was 106.6°, as shown in Fig. S1C. Similar reductions in contact angle have also been reported for other polymer-based delivery systems, including star polycation (SPc)-loaded veratramine, whose contact angle on eggplant leaves can decrease substantially to ~ 34.2° following the encapsulation, reflecting elevated affinity toward hydrophobic leaf surfaces [51]. Meanwhile, the leaf retention of TOP@HLDP was markedly improved with the aid of HLDP, and the retained mass of TOP@HLDP reached 31.48 mg/cm², compared to 13.66 mg/cm² of free TOP and 29.42 mg/cm² of commercial TOP (Fig. 3B, Fig S2D). The enhanced retention is likely due to the combined effects of reduced droplet surface tension and possible adhesive interactions arising from the cationic terminal moieties of HLDP, which promote stronger physical anchoring on the negatively-charged leaf surface [52]. Similarly, the stronger retention following HLDP complexation has been reported for some other AIs, highlighting the versatility of this nanocarrier-based delivery system in improving pesticide deposition [29, 30].

The enhancement in surface deposition of TOP@HLDP led to excellent performance of plant uptake. As shown in Fig. 3C and Fig. S2, TOP@HLDP-treated plants consistently exhibited markedly higher TOP levels in leaves than those treated with free TOP at all tested time points. Especially, the internalized content of TOP significantly increased in the presence of HLDP, with 2-fold higher uptake (404.60 mg/kg) than that of free TOP (207.60 mg/kg) at 9 h after the root application. For comparison, the plant uptake behavior of the commercial TOP formulation was also evaluated (Fig. S1E). The increased uptake is likely associated with the nanoscale size, positive-charged surface and hydrophobic structure of TOP@HLDP, which together facilitate the interactions with hydrophobic plant cuticle for translocation across the delivery barrier [53]. Consistent with previous publications, the complexation with SPc can increase the plant uptake of ABOB with significantly higher content at later time points (6–12 h) after the treatment [54].

HSI was further employed to visualize the in situ distribution of TOP and TOP@HLDP within stem tissues of *C. xanthiifolia* [55]. Enhanced dark-field hyperspectral images and corresponding false-color overlay maps were generated to compare the spatial distribution patterns of the two formulations (Fig. 3D). The stem sections treated with TOP@HLDP exhibited a broader and more continuous distribution of hyperspectral signal spots compared with those treated with TOP alone. In the TOP-treated stems, hyperspectral signals were relatively sparse and localized, whereas those treated with TOP@HLDP displayed more widely-distributed signal clusters across the stem tissue. This difference indicated that the incorporation into HLDP facilitated a more extensive dispersion of TOP within stem tissues, and the markedly-broader signal distribution provided the direct visual evidences for stronger plant uptake of HLDP-loaded TOP at the tissue scale.

The vertical soil leaching experiment revealed distinct transport behaviors between TOP and TOP@HLDP (Fig. 3E). The residual TOP of both formulations began to decline at the 3rd fraction. The TOP@HLDP rapidly approached a plateau after the 7th fraction and finally stabilized at 15.6%, whereas free TOP continued to decrease gradually and remained at 25.8% in the final fraction. These results indicated that HLDP encapsulation altered the soil transport dynamics of TOP, leading to earlier breakthrough but reduced long-tail persistence. Notably, the elution profile of TOP@HLDP reached a stable plateau after the 7th fraction, indicating that the remaining fraction was not readily mobile under the present conditions and was likely retained within the soil matrix. This behavior may be associated with the nanoscale dispersion of the formulation and its interaction with the soil matrix. From an environmental perspective, the lower residual fraction observed in the later stages might indicate a reduced tendency for long-term accumulation compared to free TOP.

### Enhanced herbicidal activity of HLDP-loaded TOP in the laboratory and field

**Fig. 4:**
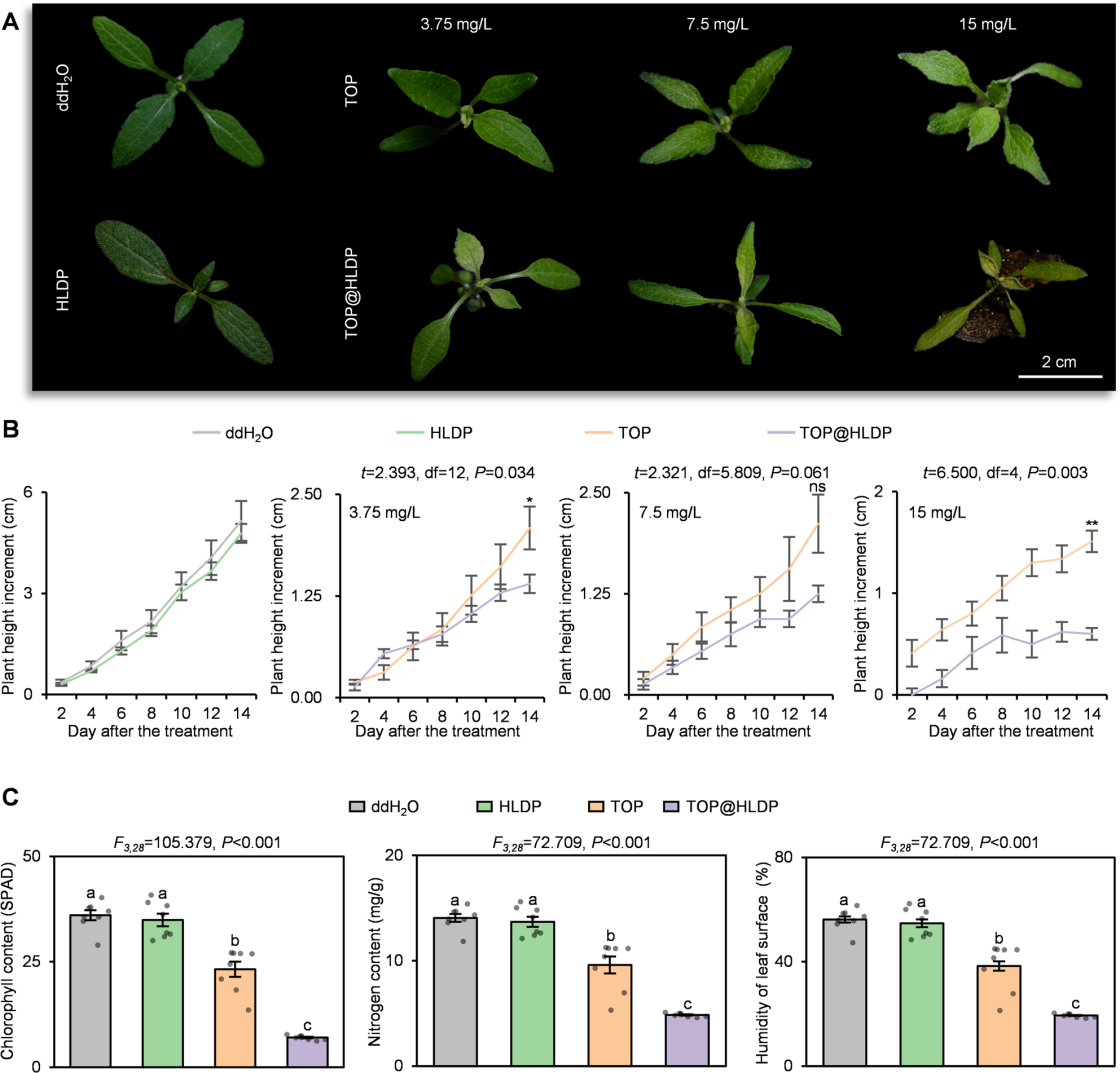
Laboratory bioassay of TOP and TOP@HLDP against *C. xanthiifolia*. (**A**) Representative photographs of *C. xanthiifolia* treated with different formulations at 7 d after the treatment. (**B**) Plant height increment of *C. xanthiifolia* treated with TOP and TOP@HLDP at the TOP concentrations of 3.75, 7.5 and 15 mg/L. Each treatment was repeated eight times. (**C**) Chlorophyll content, nitrogen content, and leaf surface moisture of *C. xanthiifolia* following different treatments. Each treatment included eight independent samples. Different letters above each bar indicate significant difference at *P* < 0.05 as determined by one-way ANOVA with Tukey HSD test

In the laboratory, the herbicidal activity of TOP@HLDP was evaluated using *C. xanthiifolia* seedlings, and the cumulative plant height increment displayed a clear divergence between the treatments of TOP@HLDP and free TOP at all tested concentrations throughout the 14-d observation period (Fig. 4A-B). For the treatment with free TOP (15 mg/L), plant height consistently increased from 2 d after treatment, reaching an average increment of 1.51 cm at 14 d after treatment. By contrast, the seedlings treated with TOP@HLDP (15 mg/L) exhibited only a slight increase during the early stage, after which growth was effectively arrested from 8 d after treatment, resulting in a final increment of just 0.50 cm at 14 d after treatment. These results indicated that the nano-herbicide exerted a more persistent inhibitory effect on weed growth. Physiological measurements of treated seedlings were consistent with their growth inhibition pattern. The chlorophyll content, nitrogen content and leaf surface moisture were the highest in seedlings treated with water and HLDP, followed by treatment with free TOP, whereas the TOP@HLDP group showed the lowest values (Fig. 4C). More specifically, the chlorophyll content, nitrogen content and leaf surface humidity decreased from 23.23 to 7.05 SPAD, from 9.60 to 4.86 mg/g, and from 38.40% to 19.43% in the seedlings treated with TOP@HLDP compared to free TOP. These results suggested that the TOP@HLDP caused a greater impairment of photosynthetic activity and nitrogen metabolism than free TOP, reflecting a stronger physiological stress response in the treated weeds. Notably, the application of HLDP alone did not affect weed growth or physiological parameters, confirming that the enhanced herbicidal activity was originated from the improved delivery of TOP rather than from intrinsic phytotoxicity of HLDP.

**Fig. 5:**
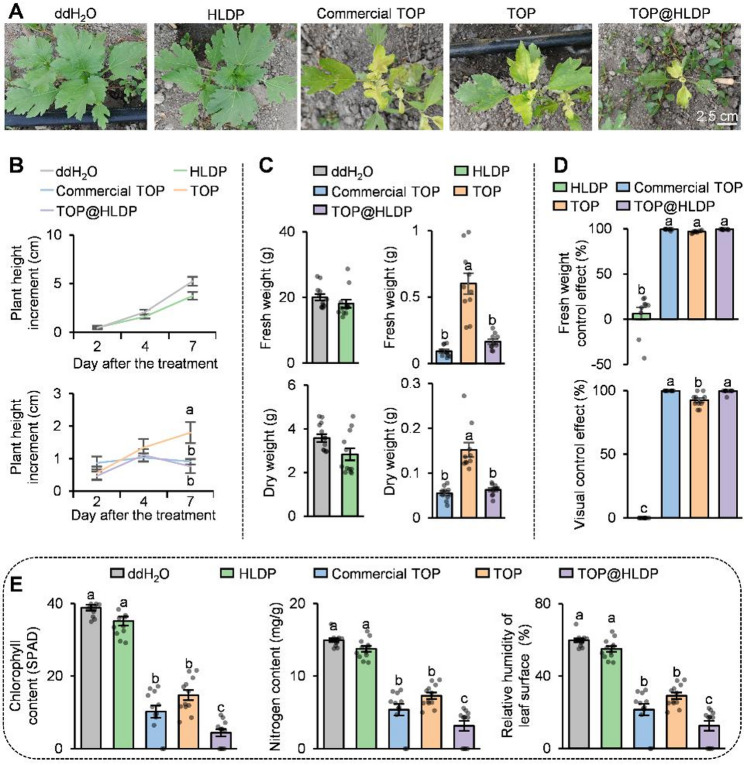
Control efficacy of TOP and TOP@HLDP against *C. xanthiifolia* in field. (**A**) Representative photographs of *C. xanthiifolia* at 7 d after the treatment. (**B**) Plant height increment of *C. xanthiifolia* at 2, 4 and 7 d after treatment. Each treatment included ten independent seedlings. (**C**) Fresh weight and dry weight of *C. xanthiifolia* at 7 d after treatment. Each treatment included ten independent seedlings. Different letters above each bar indicate significant difference at *P* < 0.05 as determined by one-way ANOVA with Tukey HSD test. (**D**) Control efficacy based on fresh weight and visual assessment at 7 d after treatment. Each treatment included ten independent seedlings. (**E**) Chlorophyll content, nitrogen content, and leaf surface moisture of *C. xanthiifolia* following different treatments. Each treatment included ten biological replicates

The field experiment further compared the herbicidal performance of TOP@HLDP with a commercial TOP (Fig. 5). The weeds treated with free TOP continued to elongate, with cumulative height increment of 1.80 cm at 7 d after treatment (Fig. 5B). By contrast, weed growth was much more strongly suppressed after the treatments with commercial TOP and TOP@HLDP, where the cumulative height increments were 0.90 and 0.77 cm, respectively. Thus, despite with only one-third of TOP concentration, the TOP@HLDP achieved excellent weed growth suppression comparable to that of commercial TOP and clearly stronger than free TOP. Biomass measurements showed a similar pattern. At 7 d after treatment, the fresh weight of weeds treated with free TOP was 0.60 g, compared with 0.16 g for TOP@HLDP and 0.09 g for commercial TOP (Fig. 5C). Consistently, the dry weights of weeds treated with free TOP, TOP@HLDP and commercial TOP were 0.15, 0.06 and 0.05 g, respectively. However, when compared to control (ddH_2_O) treatment, all three TOP-containing formulations achieved similarly high control effects, consistent with the visual control effects (Fig. 5D). Physiological measurements provided additional synergistic evidences. Severe chlorosis was observed in the weeds treated with TOP@HLDP and commercial TOP (Fig. 5E). In a proportion of leaves, the bleaching progressed to the extent that the chlorophyll and nitrogen contents decreased close to zero. This phenomenon is consistent with the complete loss of green tissues and therefore reflects advanced physiological injury. In contrast, the weeds treated with free TOP generally retained pigment levels, suggesting less severe metabolic suppression. Thus, our findings demonstrated that the self-complexation with HLDP could substantially improve the biological performance of TOP likely via enhanced foliar deposition/retention and uptake efficiency, which increased the effective herbicide dose reaching target sites within weed tissues. Similar enhancement of herbicidal performance following the complexation with HLDP has been only reported for MP, where the fresh weight control effect of MP@HLDP was estimated to be 94.0%, compared to merely 52% and 73% for free MP and commercial MP, respectively [32].

### Synergistic effect of HLDP-loaded TOP via suppressing antioxidant capacity/photosynthetic metabolism

**Fig. 6:**
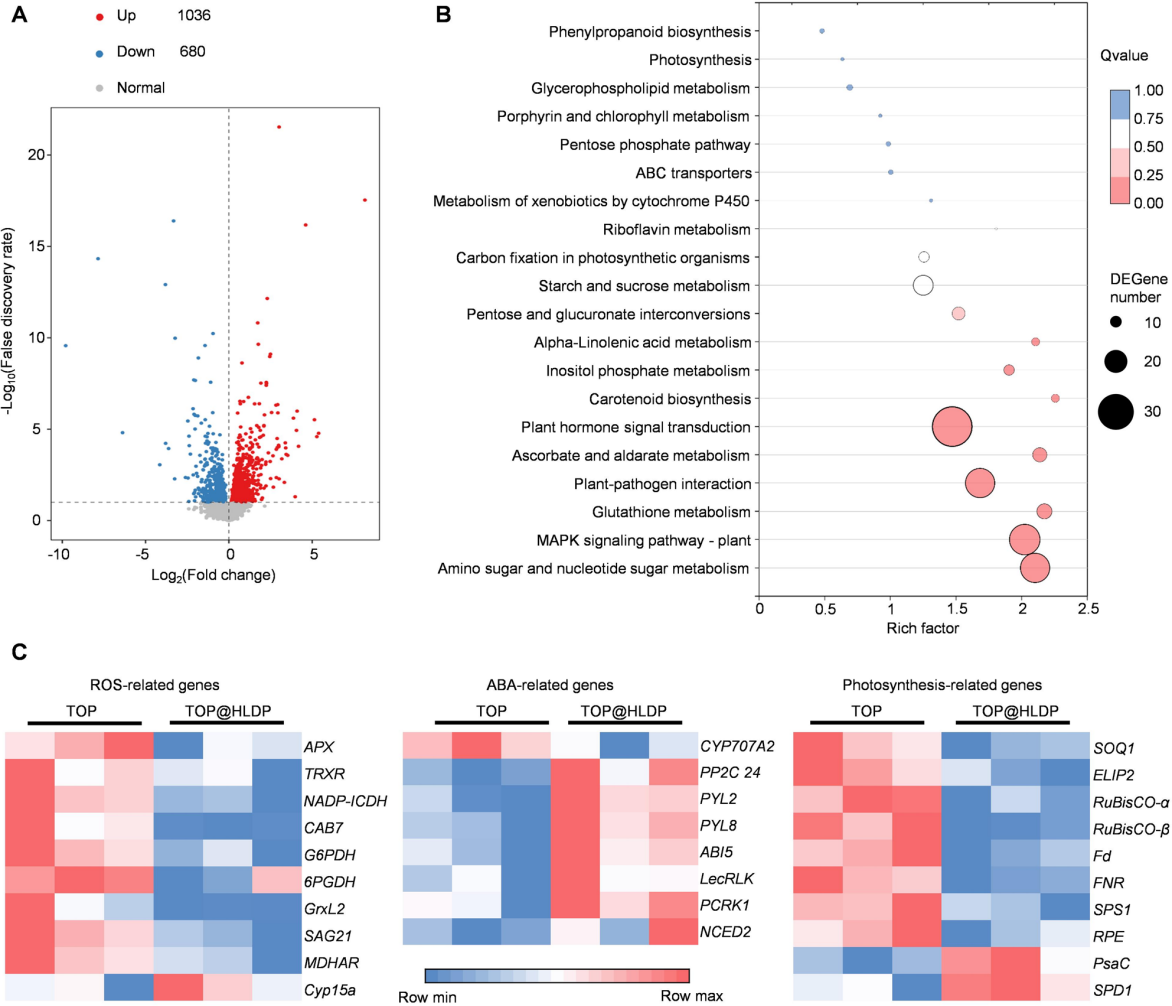
Transcriptomic analysis of *C. xanthiifolia* treated with TOP and TOP@HLDP. (**A**) Volcano plots of DEGs in leaves treated with TOP@HLDP compared with free TOP. Up- and down-regulated genes are represented by red and blue dots, respectively. (**B**) KEGG pathway enrichment analysis of DEGs. (**C**) Heat maps of DEGs associated with ROS-related pathways, ABA-related pathways, and photosynthesis-related pathways. High- and low-expression levels are indicated by red and blue colors, respectively

High-quality RNA-seq datasets were obtained for all samples (Table S2 and Fig. S3). Compared with free TOP treatment, the TOP@HLDP treatment resulted in differential expression of 1716 genes, including 1036 up-regulated and 680 down-regulated DEGs (Fig. 6A). KEGG enrichment analysis indicated that these DEGs were mainly associated with the pathways related to oxidative stress, abscisic acid (ABA) signaling, photosynthetic metabolism, etc. (Fig. 6B).

Transcriptomic profiling revealed that the application of TOP@HLDP induced a coordinated reprogramming of stress- and photosynthesis-related pathways in weeds compared to TOP alone (Fig. 6C). A prominent feature was the downregulation of key genes encoding ROS-buffering enzymes in the weeds treated with TOP@HLDP, including *ascorbate peroxidase* (*APX*), and the NADPH-generating enzymes *G6PDH*, *6PGDH* and *IDH-NADP*, together with *thioredoxin-dependent oxidoreductases* (*TRXR*). Since these enzymes collectively sustain NADPH-driven redox homeostasis, their repression suggested a weakened antioxidant buffering capacity and an increased tendency toward oxidative perturbation in the weeds treated with TOP@HLDP [56, 57]. In parallel, the ABA pathway showed clear transcriptional activation in the weeds exposure to TOP@HLDP compared to free TOP. The ABA biosynthetic gene *NCED2* was up-regulated, whereas the ABA catabolic gene *CYP707A2* was down-regulated, indicating a metabolic shift favoring ABA accumulation. At the signaling level, the expression of ABA receptor genes (*PYL2* and *PYL8*) and the downstream transcription factor *ABI5* was also induced, together supporting sustained ABA perception and stress-responsive transcription [58, 59]. These patterns are consistent with ABA–ROS crosstalk during abiotic stress adaptation, in which ABA modulates ROS production and antioxidant pathways as a part of coordinated stress signaling networks [60, 61]. Concomitantly, the genes associated with photosynthetic electron transport and carbon fixation were down-regulated, including *RuBisCO* subunits, *ferredoxin* (*Fd*) and *ferredoxin–NADP⁺ reductase* (*FNR*), implying the reduced CO₂ assimilation and chloroplast NADPH production [62, 63]. Downregulation of photoprotective regulators such as *SOQ1* further suggested a transition from efficient photochemistry toward stress-acclimatory metabolic state. Taken together, these transcriptional signatures indicated that the application of TOP@HLDP imposed stronger oxidative and hormonal stress on weeds than free TOP, thereby weakening cellular redox buffering capacity, triggering ABA-mediated stress signaling, and suppressing photosynthetic metabolism. These molecular responses were fully consistent with the enhanced physiological growth inhibition observed in the weeds treated with TOP@HLDP.

### Synergistic effect of HLDP-loaded TOP via impairing sucrose synthesis and JA utilization

**Fig. 7:**
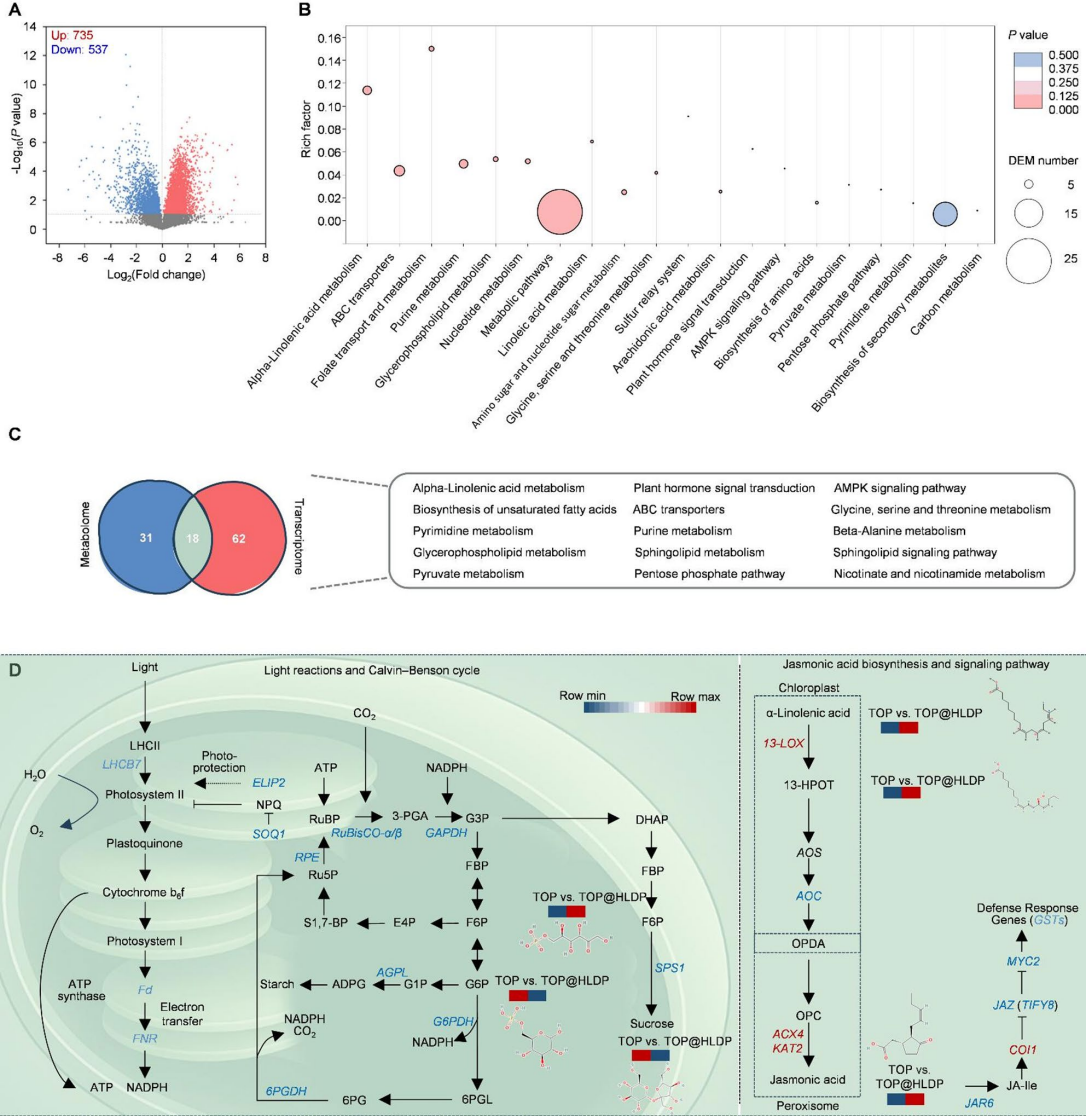
Conjoint analysis of metabolomics and transcriptomics for the synergistic mechanism of TOP@HLDP. (**A**) Volcano plots of DAMs in leaves treated with TOP@HLDP compared with free TOP. Up- and down-regulated metabolites are represented by red and blue dots, respectively. (**B**) KEGG pathway enrichment of the DAMs. (**C**) Common pathways in metabolomics and transcriptomics. Typical common pathways are provided. (**D**) Integrated pathway analysis highlighting photosynthetic light reactions, Calvin cycle, jasmonic acid biosynthesis and signaling pathways. Amounts of DAMs are indicated by heatmap using Log_2_(Fold change). Up- and down-regulated DEGs are marked in red and blue color, respectively

To further elucidate the biochemical responses of *C. xanthiifolia* to nanoscale TOP, untargeted metabolomic profiling was conducted to characterize differentially accumulated metabolites (DAMs) in the weeds treated with TOP and TOP@HLDP. Principal component analysis (PCA) showed a clear separation between two treatment groups, with tight clustering of biological replicates, confirming the robustness and reproducibility of the dataset (Fig. S4). In total, 1272 DAMs were identified, including 735 up- and 537 down-regulated DAMs in the weeds treated with TOP@HLDP, compared to free TOP (Fig. 7A). KEGG pathway annotation indicated that these metabolites were mainly associated with central metabolic processes, lipid metabolism (particularly α-linolenic acid metabolism), membrane transport (ABC transporters), and amino sugar and nucleotide sugar metabolism, suggesting the extensive reprogramming of primary metabolism, lipid-derived signaling and transmembrane transport (Fig. 7B). To integrate metabolic alteration with transcriptional regulation, a joint transcriptome–metabolome analysis was performed. A total of 18 common pathways were jointly-enriched in both transcriptomics and metabolomics, with major overlapping pathways including α-linolenic acid metabolism, plant hormone signal transduction, ABC transporters, glycerophospholipid and sphingolipid metabolism, AMPK signaling, pyruvate metabolism, etc. (Fig. 7C). The convergence of these pathways highlighted a coordinated remodeling of lipid-mediated stress signaling, hormone perception, central carbon metabolism, nucleotide turnover, and cellular redox regulation in the weeds exposure to TOP@HLDP. These multi-omic signatures provided a mechanistic basis for the stronger physiological disruption induced by TOP@HLDP compared with TOP alone.

In agreement with the transcriptomic profiles, the metabolomic dataset revealed a coordinated reprogramming of photosynthetic carbon metabolism and oxylipin biosynthesis in the weeds exposure to TOP@HLDP (Fig. 7D). Integrated pathway mapping showed that the key components for light reactions, including *LHCB7*, *Fd* and *FNR*, were markedly down-regulated in the weeds treated with TOP@HLDP, indicating impaired electron transfer from PSI and reduced NADPH generation [64]. Meanwhile, the suppression of photoprotective regulators such as *ELIP2* and *SOQ1* suggested the altered regulation of non-photochemical quenching and a weakened capacity for light acclimation [65, 66]. Consistent with disrupted electron transport, metabolites in the Calvin cycle and starch–sucrose pathway exhibited distinct accumulation patterns in the weeds treated with TOP@HLDP: glucose-6-phosphate (G6P) was significantly depleted, whereas fructose-6-phosphate (F6P) accumulated, accompanied by the down-regulation of *sucrose-phosphate synthase* (*SPS1*) and a marked reduction in sucrose content. These changes indicated a metabolic bottleneck in sucrose synthesis and redistribution of carbon flux away from assimilatory metabolism, while simultaneously limiting substrate supply to the pentose-phosphate pathway (PPP) and thereby reducing NADPH availability for ROS detoxification [67–69].

Conjoint metabolomic–transcriptomic analysis further revealed that TOP@HLDP treatment reshaped jasmonate (JA) metabolism and signaling in weeds (Fig. 7D). The contents of precursor metabolite α-linolenic acid and 13-HPOT were lower in the weeds treated with TOP@HLDP compared to TOP alone, and key biosynthetic genes including *13-LOX*, *ACX4* and *KAT2* were up-regulated, together with a marked increase in endogenous JA content, indicating stronger JA biosynthesis under sustained stress [70]. However, several downstream signaling components, such as *JAR6*, *JAZ* (*TIFY8*) and *MYC2*, were transcriptionally down-regulated in the weeds treated with TOP@HLDP compared with TOP alone. This pattern suggested that although JA production was activated at the metabolic level, the transcriptional program governing JA-responsive defense was concurrently attenuated, consistent with the observed reduction in GST-mediated detoxification and antioxidant capacity [71–73]. Overall, the integrated transcriptomic–metabolomic evidences pointed to a broader physiological context in the weeds exposure to TOP@HLDP, in which chloroplastic NADPH generation was weakened, carbon allocation was rerouted away from assimilatory metabolism, and hormonal stress responses became increasingly uncoupled. Such disturbances in metabolic and signaling homeostasis provided a coherent explanation for the stronger oxidative stress and growth suppression observed in the weeds exposed to TOP@HLDP. This uncoupling between enhanced JA biosynthesis and suppressed downstream signaling might impair the coordination of defense and detoxification processes, thereby exacerbating oxidative stress and contributing to growth inhibition.

### Severe disruption of oxidative response and carotenoid synthesis in the weeds treated with TOP@HLDP

**Fig. 8:**
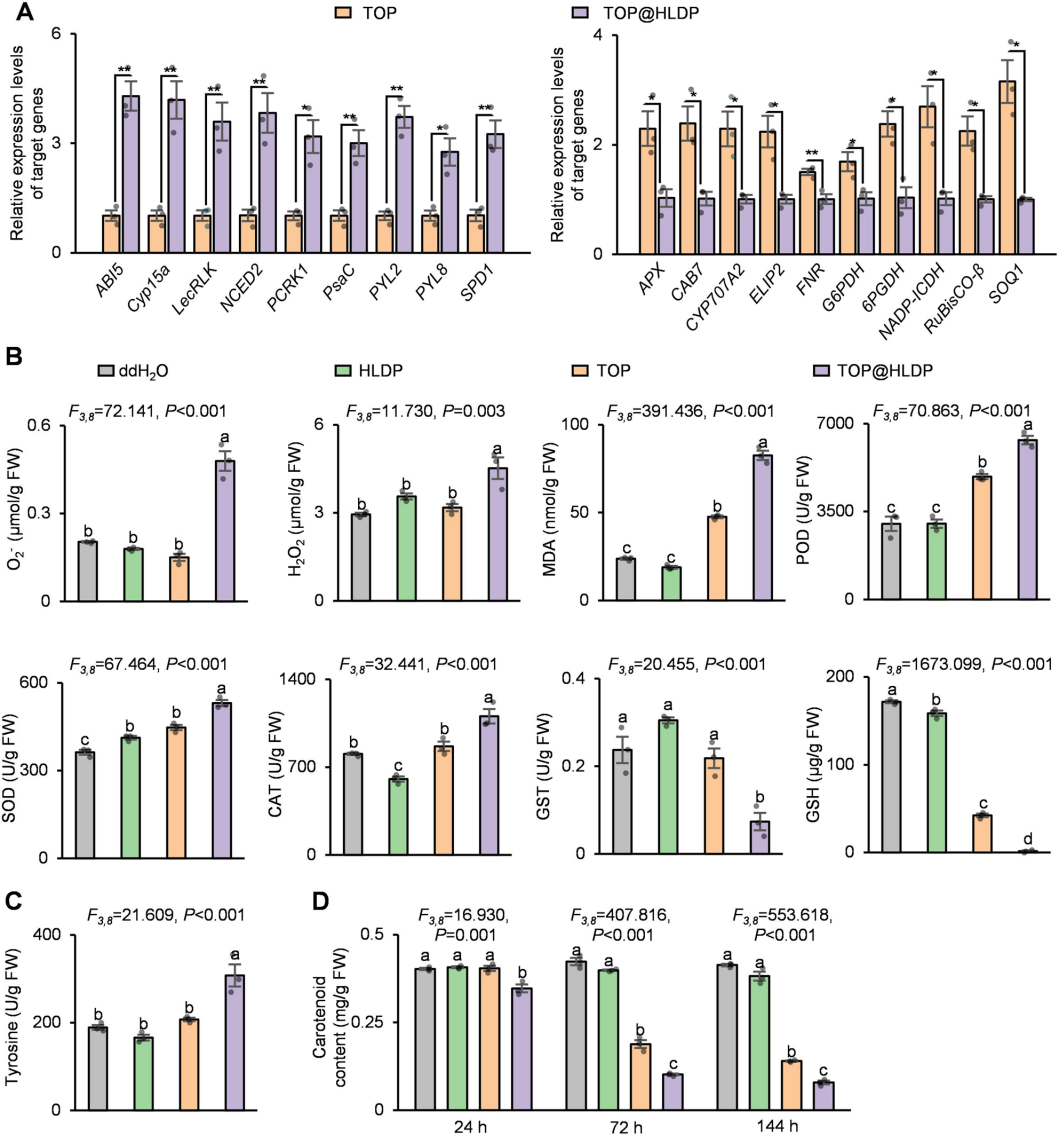
Gene expression validation and physiological responses of *C. xanthiifolia* treated with TOP and TOP@HLDP. (**A**) Validation of DEGs using qRT-PCR. Each treatment contained three independent samples, and the asterisk indicated significant difference according to independent *t*-test (*P < 0.05 and **P < 0.01). (**B**) Levels of O₂⁻, H₂O₂, MDA, and activities of POD, SOD, CAT, GST, as well as GSH content in weeds treated with various formulations. Each treatment contained three independent samples. Different letters above each bar indicate significant difference at *P* < 0.05 as determined by one-way ANOVA with Tukey HSD test. (**C**) Tyrosine content in weeds treated with various formulations. Each treatment contained three independent samples. (**D**) Carotenoid content in weeds at 24, 72 and 144 h after treatment with various formulations. Each treatment included three biological replicates

To verify the reliability of RNA-seq results, nineteen DEGs were firstly selected for qRT-PCR validation. The expression patterns of tested genes were highly consistent with the transcriptomic results, confirming the robustness of the sequencing data (Fig. 8A).

Consistent with the transcriptome–metabolome signatures, the physiological indices further demonstrated that the TOP@HLDP triggered markedly stronger oxidative burst and target inhibition than free TOP (Fig. 8B). The application of TOP@HLDP resulted in the highest accumulation of both O₂⁻ and H₂O₂, reaching 0.48 and 4.52 µmol/g FW, respectively. This indicated that the TOP@HLDP imposed the most intense oxidative burden on weeds, in agreement with the enhanced ROS production reported under herbicide-induced stress [74, 75]. Meanwhile, the antioxidant system was insufficient to counterbalance ROS over-accumulation, as evidenced by the highest MDA content (82.55 nmol/g FW), a reliable biomarker of lipid peroxidation and membrane injury [76]. These data collectively suggested that TOP@HLDP exposure elicited the most severe oxidative damage. In response, most core ROS-scavenging enzymes exhibited a hyper-induction pattern, and SOD (531 U/g FW), CAT (1106 U/g FW) and POD (6346 U/g FW) all reached the highest activities in the weeds treated with TOP@HLDP. Such up-regulation reflects strong oxidative signal perception and activation of enzymatic detoxification network, which converts highly reactive ROS into less harmful forms to maintain redox balance [76, 77]. However, reduced GST activity and GSH content were observed in the weeds treated with TOP@HLDP compared with TOP alone.

The physiological profiles also corroborated the enhanced target inhibition in the weeds treated with TOP@HLDP. As an HPPD-inhibiting herbicide, TOP usually blocks the conversion of tyrosine, leading to precursor accumulation. The TOP@HLDP treatment resulted in the highest tyrosine content in weeds among all groups, indicating that the nanoscale TOP achieved a more rapid and efficient enzymatic blockade than free TOP (Fig. 8C). This enhanced biochemical inhibition was accompanied by a pronounced decline in carotenoid content, which ultimately reached the lowest level (0.08 mg/g FW) at 144 h after the treatment with TOP@HLDP (Fig. 8D). Since carotenoids act as photoprotective antioxidants that quench triplet chlorophyll and singlet oxygen, their depletion weakens the defense against photooxidative stress and accelerates chloroplast injury [75, 78]. Thus, the TOP@HLDP intensified both biochemical target inhibition and oxidative collapse, producing a synergistic effect in phytotoxicity. Taken together, these physiological data demonstrated that the TOP@HLDP induced a dual-phase stress pattern: (i) a strong ROS burst triggering transient hyper-activation of antioxidant enzymes, followed by (ii) exhaustion of the GSH–GST detoxification system and irreversible oxidative membrane damage. This biochemical failure, combined with rapid HPPD inhibition and carotenoid depletion, provided strong mechanistic evidences for the superior herbicidal activity of TOP@HLDP.

### Enhanced biosafety of HLDP-enabled TOP toward non-targets

**Fig. 9:**
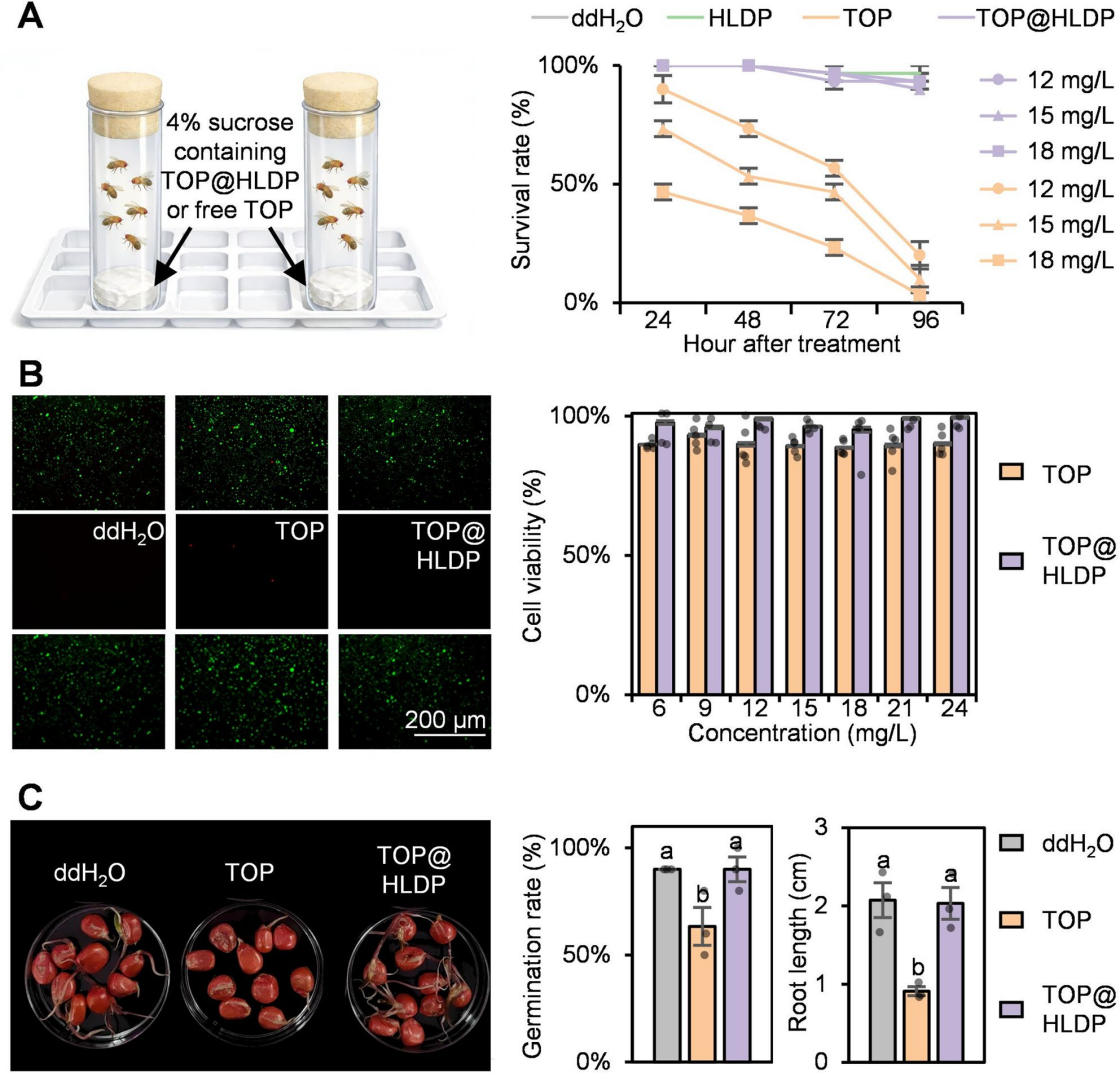
Biosafety evaluation of TOP and TOP@HLDP toward non-target organisms. (**A**) Survival rate of fruit fly adults following exposure to TOP and TOP@HLDP. Each treatment contained twenty adults, which was repeated three times. (**B**) Cytotoxicity assessment of TOP and TOP@HLDP toward S2 cells, visualized by alive/dead cell fluorescence staining. Each treatment was repeated five times. (**C**) Germination rate and root length of maize seeds treated with TOP and TOP@HLDP. Each treatment included ten seeds, which was repeated three times. Different letters above each bar indicate significant difference at *P* < 0.05 as determined by one-way ANOVA with Tukey HSD test

The biosafety of TOP@HLDP was assessed using the fruit flies, S2 cells and maize seeds. At 96 h post-exposure, free TOP exhibited severe acute toxicity toward the adults of fruit flies, with the mortalities of 80.00%, 90.00% and 96.67% at concentrations of 12, 15 and 18 mg/L, respectively, indicating a pronounced concentration-dependent toxic effect even at field-relevant concentrations (Fig. 9A). As expected, the treatment with ddH_2_O (control) or HLDP showed negligible adverse impacts on fruit flies. Notably, the encapsulation within HLDP dramatically reduced insect mortality, and the oral feeding of TOP@HLDP resulted in low mortalities of only 6.67%, 10.00% and 6.67% at the corresponding concentrations. Consistent with above assessment, the cellular biosafety of HLDP-loaded TOP was further improved toward S2 cells across a concentration range of 6–24 mg/L (Fig. 9B). Free TOP caused a moderate but consistent reduction in cell viability, with relative viabilities ranging from 89% to 93% across the tested concentrations, indicating a certain level of cytotoxic stress. In contrast, TOP@HLDP treatment maintained significantly higher cell viability of 95%-99%. The striking reduction in acute toxicity and improved cellular tolerance observed for TOP@HLDP suggested that the encapsulation within HLDP could effectively alleviate the direct exposure to TOP, thereby reducing its larvacidal/cytotoxic effects. This phenomenon is consistent with previous reports on polymer-based nano-formulations, in which nanocarrier-mediated modulation of bioavailability and cellular uptake can enhance cytocompatibility compared with free AIs. For example, nano-pesticides have been reported to exhibit 43.1% lower toxicity toward non-target organisms compared with conventional formulations [79]. The β-CYP/ZIF-8 nano-formulation displays markedly improved biosafety toward human hepatocyte LO2 cells in cytotoxicity assays [80].

To further assess crop-level biosafety, the effects of free TOP and TOP@HLDP on germination and seedling growth of maize seeds were evaluated at the field-relevant concentration of 15 mg/L (Fig. 9C). The treatment with TOP alone significantly impaired the germination and growth of maize seeds, which resulted in a lower germination rate of 63.33% and a markedly shortened primary root length of 0.91 cm, compared to the control (90.00% germination and 2.07 cm root length). In contrast, TOP@HLDP exhibited negligible adverse effects on maize seeds, as both the germination rate (90.00%) and root length (2.03 cm) were comparable to those of the control group. These results indicated that the complexation with HLDP effectively alleviated the phytotoxicity of TOP during early plant development, likely by reducing its direct exposure of germinating tissues. Similar alleviation of herbicide-induced crop stress has been reported for polymer-based nano-formulations, where nanocarrier-mediated controlled release/buffering can preserve the crop safety while maintaining herbicidal efficacy [81].

## Conclusion

In this study, the polymeric nanocarrier HLDP was employed to construct a nano-enabled herbicide (TOP@HLDP), and the self-assembly was achieved via multiple interaction forces between HLDP and TOP, including hydrophobic association and hydrogen bonding. The resulting TOP@HLDP formed nanoscale spherical or near-spherical particles with smooth surfaces and uniform morphology, and the complexation with HLDP improved the thermal stability of TOP by forming a hybrid nanostructure. Notably, the wetting and adhesion behaviors of TOP@HLDP were remarkably improved on weed leaves, and its enhanced droplet deposition resulted in stronger plant uptake and systemic transport. Interestingly, soil leaching tests revealed that TOP@HLDP exhibited altered transport behavior compared to free TOP, with earlier elution and a lower residual fraction in the later stages, suggesting differences in soil mobility and retention characteristics. Importantly, the TOP@HLDP displayed stronger herbicidal activity toward *C. xanthiifolia*, with the lower contents of chlorophyll and nitrogen compared with free TOP in both laboratory and field. Integrated transcriptomic and metabolomic analyses demonstrated that the HLDP-based delivery intensified TOP-induced redox imbalance and photosynthetic disruption in weeds, thereby impairing the sucrose synthesis and JA utilization to aggravate the growth inhibition. Furthermore, the TOP@HLDP showed favorable biosafety toward non-target organisms. Overall, our results demonstrated that the HLDP could be applied as an efficient amphiphilic block copolymer/adjuvant to improve the delivery/bioactivity of TOP, which provided a strategy for enhancing herbicidal efficacy through improved delivery and intensified stress responses.

## Electronic Supplementary Material

Below is the link to the electronic supplementary material.

Supplementary Material 1.

## Data Availability

The data that support the findings of this study are available from the corresponding author upon reasonable request.
