# Supplementary material for: Multilevel synergistic mechanisms of a nanocarrier-enabled topramezone herbicide against invasive weeds
Source: J Nanobiotechnology. 2026 Apr 22;24:541. doi: 10.1186/s12951-026-04472-5 (PMC13251031; doi:10.1186/s12951-026-04472-5)
Supplement: Supplementary file 1 — Supplementary Material 1. [file 12951_2026_4472_MOESM1_ESM.docx]

**Appendix A. Supplementary data**

**Multilevel Synergistic Mechanisms of a Nanocarrier-Enabled Topramezone Herbicide against Invasive Weeds**

**Jingyi Chen^1^, Heng Qiao^1^, Xiao Ran^1^, Jixing Xia^1^, Hegan Dong^2^, Hanyue Wang^2^, Huan Peng^3,4^, Meizhen Yin^5^, Min Dong^1^, Jie Shen^1^ and Shuo Yan^1*^**

^1^State Key Laboratory of Agricultural and Forestry Biosecurity, Department of Plant Biosecurity, College of Plant Protection, China Agricultural University, Beijing 100193, PR China

^2^College of Life Sciences in Shihezi University and Xinjiang Production and Construction Corps Key Laboratory of Oasis Town and Mountain-basin System Ecology, Shihezi 832003, PR China

^3^Institute of Western Agriculture, Chinese Academy of Agricultural Sciences, Changji 453500, PR China

^4^State Key Laboratory for Biology of Plant Diseases and Insect Pests, Institute of Plant Protection, Chinese Academy of Agricultural Sciences, Beijing 100193, PR China

^5^State Key Laboratory of Chemical Resource Engineering, Beijing Lab of Biomedical Materials, Beijing University of Chemical Technology, Beijing 100029, PR China

^*^Correspondence: yanshuo2011@foxmail.com


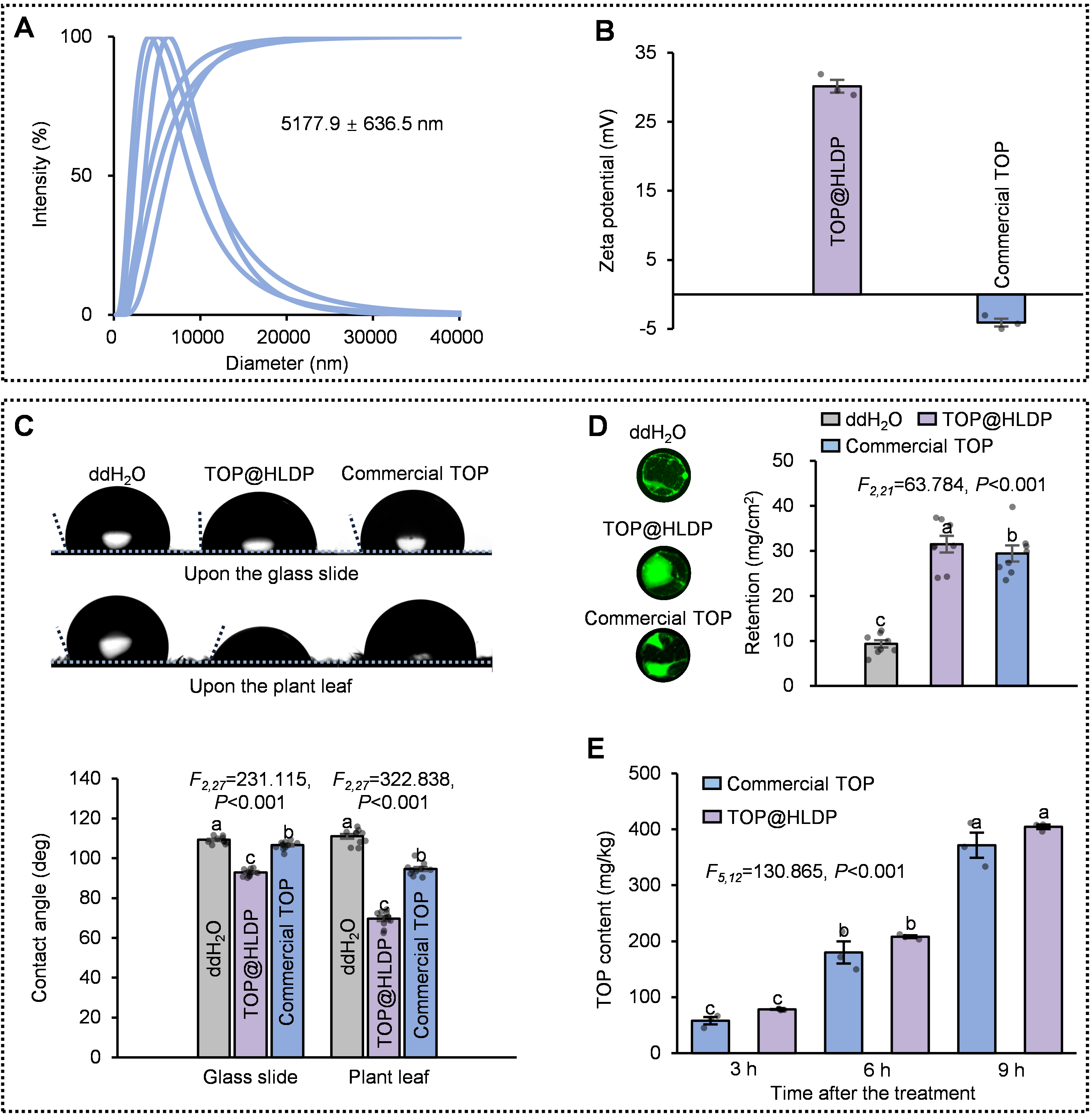


**Fig. S1.** Physicochemical characterization and foliar behavior of commercial TOP. (A) Hydrodynamic diameter of the commercial TOP formulation. Each solution was tested three times. (B) Zeta potential of the commercial TOP. Each solution was tested three times. (C) Contact angles of the commercial TOP on the leaf surface of *C. xanthiifolia*. Each formulation was tested ten times. Different letters above each bar indicate significant difference at *P* < 0.05 as determined by one-way ANOVA with Tukey HSD test. (D) Retention of the commercial TOP on the leaf surface of *C. xanthiifolia*. Each treatment was repeated eight times. (E) Uptake of the commercial TOP by *C. xanthiifolia*. Each treatment was repeated three times.


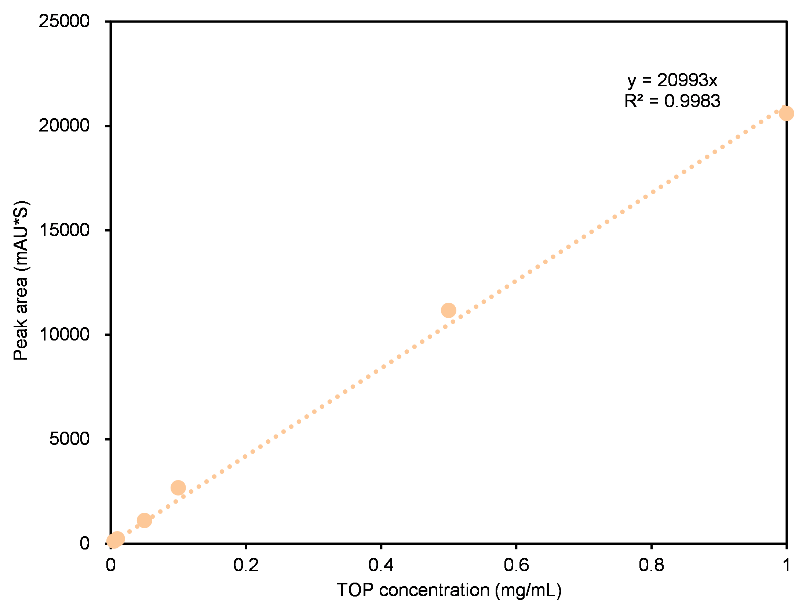


**Fig. S2.** Calibration curve of TOP determined by high-performance liquid chromatography (HPLC).


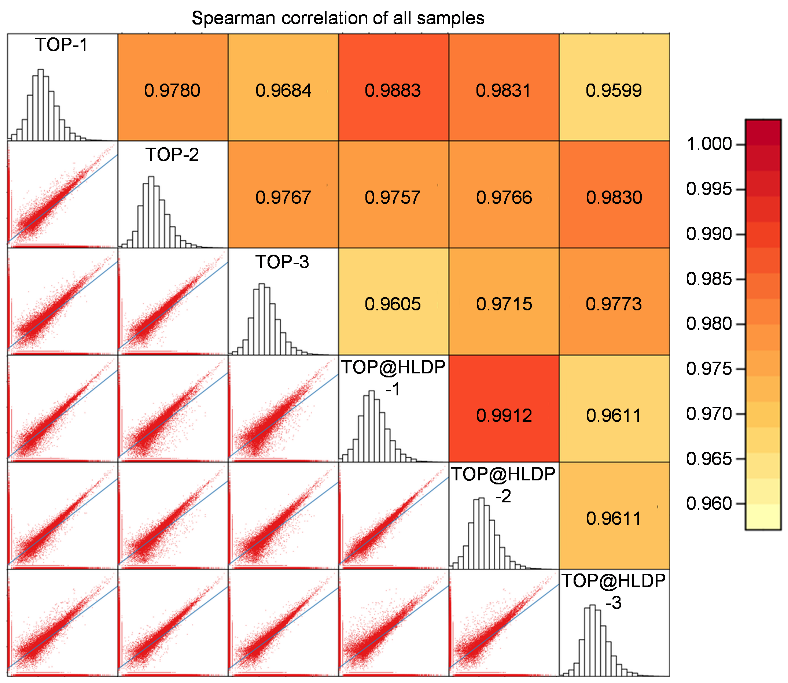


**Fig. S3.** Spearman correlation between collected samples. The TOP-1, TOP-2 and TOP-3 indicate TOP treatment, and TOP@HLDP-1, TOP@HLDP-2 and TOP@HLDP-3 indicate TOP@HLDP treatment.


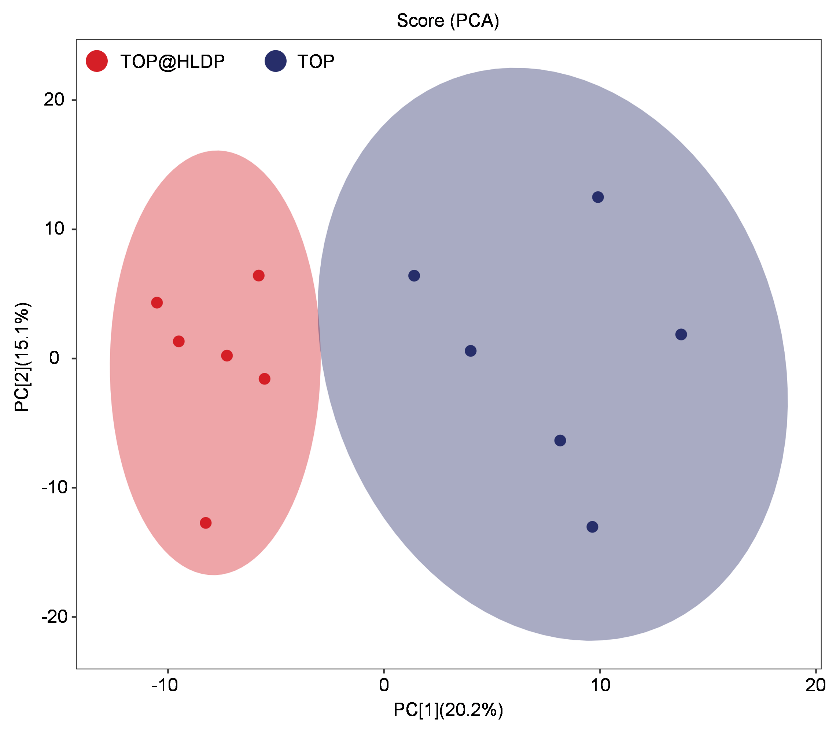


**Fig. S4.** Principal component analysis (PCA) score plot of metabolomic profiles.

**Table S1. Primers for quantitative real-time PCR.**

| **Target gene** | **Primer** | **Sequence (5’-3’)** |
| --- | --- | --- |
| *EF1α* | EF1α-F | CGATTTCCGGATTCGAGGGT |
|  | EFα-R | GCTTGTCGGAGGGTCTCTTC |
| *ABI5* | ABI5-F | ACTGCCTTGCAACACGGATA |
|  | ABI5-R | GAATCCAAAGATGCCGTCGC |
| *Cyp15a* | Cyp15a-F | TCGGCGTAGGTTAGCCTTGA |
|  | Cyp15a-R | GTCCCAACCGAAGAAACACCT |
| *LecRLK* | LecRLK-F | GAGAGAATTTGGAGAGGCAAGG |
|  | LecRLK-R | CTCTCACCATTCACCAACACAT |
| *NCED2* | NCED2-F | ACACGTTCCAGTTTTGTTGGTT |
|  | NCED2-R | ATTCGATCGTTTCCGCGCTT |
| *PCRK1* | PCRK1-F | TCTGGCTCTCATGCTACTGC |
|  | PCRK1-R | CGTGCAGGCCCATTTTCTTG |
| *PsaC* | PsaC-F | TCCAAAGCCACAGGATACGAC |
|  | PsaC-R | GGCGGGAGGTTCCATTTTTC |
| *PYL2* | PYL2-F | AGGATTTTGATGGGGTCGTGT |
|  | PYL2-R | GAAGACGCGGACGCAAATGA |
| *PYL8* | PYL8-F | CTCCAACGAGTCTGTAGCGT |
|  | PYL8-R | ACGAATCAACCGACTCTGTGAC |
| *SPD1* | SPD1-F | TGTGTGTCACCCTGTAAGTCA |
|  | SPD1-R | CAACTGCAATGGGTTGTTTGG |
| *APX* | APX-F | CAACCGGATAGCACTTCCCC |
|  | APX-R | CATCCTAGGGTTTCTCGCTCG |
| *CAB7* | CAB7-F | AACTACCACTCCAGCAACCTC |
|  | CAB7-R | GCTTCATGGCAAATCACGAC |
| *CYP707A2* | CYP707A2-F | CACTCCCTCCCTCCAAACTT |
|  | CYP707A2-R | TTCCATGTTGGTTGGTTTGGTT |
| *ELIP2* | ELIP2-F | AGCGGCCAAGGTGTCTTATC |
|  | ELIP2-R | GACTTGGACTCTGCCCTGAC |
| *FNR* | FNR-F | ACTAGCATGGCATCTGCGTAA |
|  | FNR-R | ACAAGCCAAACTCGGCCA |
| *G6PDH* | G6PDH-F | CCATCTTGCAGATGAATGGCT |
|  | G6PDH-R | TCTTCATCTACACATTGTCCTGCT |
| *6PGDH* | 6PGDH-F | CATGTTGGAGTGGAGAGGGG |
|  | 6PGDH-R | TCACTTCATCGACGAGTCCG |
| *NADP-ICDH* | ICDH-F | GTTTTAACCCCGCACGACCA |
|  | ICDH-R | AAGACGGACCAAACGGCTTA |
| *RuBisCO-β* | RuBisCO-β-F | GTGGAGCCTGTTTGAGAGCA |
|  | RuBisCO-β1R | TTCCAGTAACAGCAGGGTGA |
| *SOQ1* | SOQ1-F | CCTCTTGGCATGCATCAGGT |
|  | SOQ1-R | CCCGGCAACTAAGAGGTTGT |

**Table S2. Sequencing quality and genome mapping for RNA-seq analysis.**

| **Sample** | **Clean reads** | **Q20** | **GC percent** | **Percentage of mapped reads** |
| --- | --- | --- | --- | --- |
| TOP-1 | 25950971 | 99.45% | 43.59% | 79.93% |
| TOP-2 | 23385212 | 99.47% | 43.74% | 79.87% |
| TOP-3 | 21263442 | 99.46% | 43.47% | 80.72% |
| TOP@HLDP-1 | 23373846 | 99.45% | 43.67% | 79.79% |
| TOP@HLDP-2 | 23144659 | 99.48% | 43.68% | 79.79% |
| TOP@HLDP-3 | 25606190 | 99.46% | 43.52% | 79.56% |
